# Health System Stakeholders’ Perspective on the Role of Mobile Health and Its Adoption in the Swiss Health System: Qualitative Study

**DOI:** 10.2196/17315

**Published:** 2020-05-11

**Authors:** Myriam Lingg, Verena Lütschg

**Affiliations:** 1 SOS Beobachter Foundation Zurich Switzerland; 2 About Tomorrow Consulting Zurich Switzerland

**Keywords:** mobile health, mHealth, eHealth, telehealth, telemedicine, digitization, electronic health record, technology

## Abstract

**Background:**

Digital health solutions have great potential to change the way health care is delivered, including better clinical outcomes and improved processes and access to health services. However, the adoption of mobile health (mHealth) solutions for patient monitoring has been rather slow in Switzerland. The reasons are complex, and a better understanding is needed to leverage the full potential of mHealth.

**Objective:**

This study aimed to deepen the understanding of the potential relevance and influence of mHealth for the health system and health care provision, and factors influencing its adoption. The findings will be used to provide an outlook on feasible recommendations for action.

**Methods:**

We conducted a qualitative survey using a maximum variation sample of a heterogeneous group of stakeholders (N=50) in the Swiss health care system with a profound knowledge of digital health and medical devices. A semistructured interview guide including open- and closed-ended questions was used to address questions around mHealth relevance and its influence on the health system, the relevance of selected determinants for mHealth adoption, and important influencing factors. A content analysis method was applied.

**Results:**

Overall, respondents thought that mHealth would have a beneficial impact on the Swiss health system but that its adoption would evolve slowly. We derived 23 key opportunities regarding patient and patient pathway, treatment of disease, and diseases and health conditions. High consistency in answers among respondents was observed for *treatment of disease*. Stakeholders’ attitudes toward mHealth adoption along the relevance of 23 preselected determinants were relatively consistent. However, we obtained diverging attitudes regarding the influence of *trends*, *enablers*, and *restraints* in Switzerland and translated them into 26 key themes influencing mHealth adoption. Relevant trends comprise *changing needs and expectations of patients*, *a rising need for efficient health care delivery*, *growing interest in improved outpatient care*, and *emerging technologies and progressing digitization*. Important enablers include *growing demand for new financing schemes and incentive concepts*, *rising demand for comprehensive information on and stronger body of evidence for mHealth use cases*, and *increasing need for easy to use alternate care approaches*. Challenging restraints are *rigidness of thinking and siloed actions of health system actors*, *complexity of changing the existing regulations and structures*, *little understanding of mHealth use and the role of clinicians*, and *risk of further polarization of the population*.

**Conclusions:**

This study provides a comprehensive look at mHealth in the Swiss health system. It becomes apparent that strong governance is inevitable to foster a sustainable data strategy and to reconcile the different interests of stakeholders. The use of mHealth will add value but will not necessarily reduce the burden on the system caused by emerging societal needs and changing disease prevalence.

## Introduction

### Background

Digital health solutions have great potential to change the way health care is delivered. This includes better clinical outcomes as well as improved processes and access to health services. As shown in literature, the promise of deploying digital solutions adds value for the patient, provider, and payer [1-5]. Use cases range from remote patient coaching, monitoring, diagnostics, prognosis, and adherence management (patient compliance) to processes that take place between health care providers.

Mobile health (mHealth), a fast-growing field in digital health, is a wireless mobile health app or device that can be used to support different phases of the patient journey [5]. It refers to the collection methods of personal health data (eg, by sensor technology) and their translation into comprehensive information (eg, artificial intelligence-enabled data analysis). The versatile opportunities provide doctors and patients with new insights regarding the patient´s real-time health status or progress of disease. This allows for immediate action and more personalized recommendations [4,5].

The digitization strategy of the Swiss health policy fosters digitization in the health system, comprising important issues which aim to advance, for example, the information technology (IT) infrastructure and adoption of digital solutions such as mHealth [6,7]. However, the adoption of digital solutions and services, according to recent studies, is progressing slowly [8,9]. Today, agreed aims and actions at all governance levels still show neither substantial impact on the hospital landscape nor the clinical practice in primary care [10-12]. However, during health care expert panels and conferences in Switzerland, it is increasingly pointed out where framework conditions have advanced and may allow next digitization steps.

In many countries, mHealth technology is a fast-developing field [13], but good practices in health care to promote its adoption are scarce [14] and the different expectations and needs of multiple stakeholders involved are rarely sufficiently aligned [15]. The adoption depends on an interplay of a complex set of either enabling or hindering factors such as trust of professional end users, administrators, and patients in digital health solutions [16]. Necessary adjustments in the different health system levels along legal, regulatory, technological, and operational dimensions also fall into these factors [1,7,8,15].

Researchers and experts have described relevant themes concerning mHealth adoption in the Swiss health system, thus contributing to a better understanding. These themes include (1) essential fields of action along the different governance levels as mentioned in literature and summarized in Table 1 [5,6,14,17,18], (2) the present relevance and usage of solutions supporting digital health by health care providers and patients [19-21], and (3) needs and requirements of clinicians regarding mHealth use [16,22-24]. Less well explored is the potential mHealth adoption from an integrated perspective of multiple stakeholders that provide health care or shape health care provision.

**Table 1:** Essential fields of action along with the different governance levels.

Area of action	Aim	Important actors
Legal framework for the use of mobile health solutions	Regulation of liability risks, a demarcation between a lifestyle and medical device app.	Authority (regulatory, policy, and normative); Associations of health care providers
Data privacy and safety	Regulate data transfer and permissions to the data, access rights, permissibility of data transfer to third parties, storage location, and liability issues.	Mobile health developer; normative authorities
Evidence of mobile health solutions	Creating trust in mobile health solutions by certification and proof of evidence of mobile health solutions.	Associations of health care providers; mobile health developers
Reimbursement for the use of mobile health	Services associated with the use of mobile health must be appropriately included in the tariff and reimbursement catalog. Similarly, virtual consultations should be billable via mobile health apps or online platforms.	Regulatory authority, associations of health care providers
Interoperability of mobile health apps	Implementing the use of mandatory standards for the interoperability of mobile health solutions and devices as an important prerequisite for realizing the potential of mobile health.	Authority (normative and policy); Experts in medical informatics and IT^a^; health care providers; mobile health developers
Enabling potential mobile health users	Stepwise introduction of digital tools for health care provision contexts and training in the use of digital health solutions.	All stakeholder groups

^a^IT: information technology.

### Objective

This study aimed to deepen the understanding of potential mHealth adoption in the Swiss health system. To achieve this aim, we assess and evaluate stakeholder perspectives regarding the potential relevance and influence of mHealth for the health system and health care provision, and factors influencing its adoption. The findings will be used to provide an outlook on feasible recommendations for action.

## Methods

### Study Design

**Figure 1:**
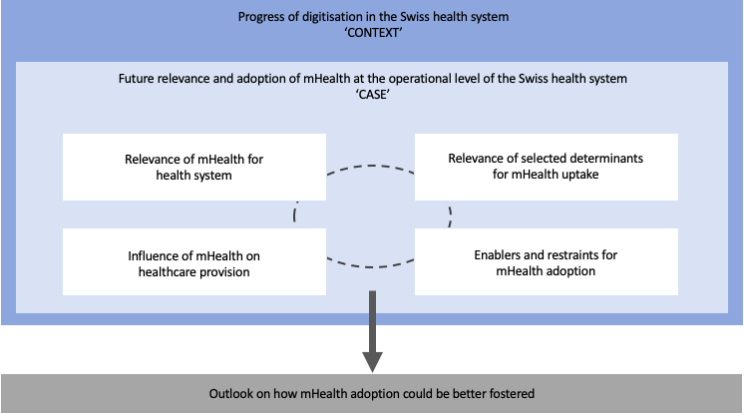
Embedded case study methodology.

We used an embedded case study methodology to integrate quantitative and qualitative methods into a single research study [25,26] by utilizing multiple sources of stakeholders to broaden and deepen data collection, bring together a wealth of data through triangulation and contribute to the validity of the research. These methods helped gain rich insights for this study that focuses on a multifaceted understanding of the future mHealth adoption in the Swiss health system by approaching the same issue from different angles. We have illustrated the main considerations of this approach in Figure 1 which will guide our data collection and analysis.

We assessed the attitudes of different health system stakeholders toward future mHealth adoption at the operational level of the Swiss health system (*case*). We did this against the background of the progress of digitization in the Swiss health sector (*context*). Further, we based the study design on 4 embedded subunits (*units of analysis*) to answer the study objectives. This gave us the opportunity for a more differentiated analysis.

### Sampling Technique and Participants’ Profiles

We used a maximum variation sample [27,28] concentrating on stakeholders that provide health care or shape health care provision, and we applied 2 selection criteria: (1) recruitment of a heterogeneous sample across stakeholders concerned with digital health topics (clinicians, health care organizations, pharmacy, medical device industry, health care start-ups, health sector associations, experts in medical informatics and IT, digital health-related experts, reimbursement-related actors, and government- and research-related bodies); and (2) stakeholders with the ability to provide rich and in-depth information about digitization, electronic health (eHealth), and mHealth, medical device regulations, and reimbursement. We conducted the study with stakeholders from Switzerland and identified interview participants by first searching listings from websites of authorities and similar bodies, Swiss health care start-ups, health technology suppliers, health care organizations, and pharmacies. We then identified authors of reports on eHealth and mHealth policy regulation, regulation and use in Switzerland, and finally, we asked interviewees to recommend other stakeholders. We contacted prospective interview participants by email or LinkedIn between July and September 2019.

### Sample Size and Data Collection Method

In total, 50 interviews were conducted between July and October 2019 by the principal investigator and a research assistant (Table 2).

Interviewees had a choice of being interviewed in German or English. We used a file naming system and anonymized interviewees by generating a list of archival numbers. We conducted face-to-face (n=38) or phone interviews (n=11), and 1 follow-up phone call was based on written participation (n=1). Interviews averaged 36 min (range 23-59 min). Interviews were not audio-recorded, but detailed notes were taken. Written consent was given by all participants, and monetary or other compensation for participation was not provided.

We used a semistructured interview guide. The selection of questions was guided by the experience of the investigators in health systems, health technologies, and digital health. We used the themes of Table 1 and a literature search as an orientation to define the open-ended questions and to select determinants for the closed-ended questions. The guide was based on 4 sets of questions: (1) what is the potential relevance of mHealth for the Swiss health system, (2) what is the potential influence of mHealth on health care provision, (3) what is the relevance of selected determinants for mHealth adoption, and (4) what are the influencing factors for mHealth adoption. We selected relevant topics for the operational level where health care services are provided to patients.

Before data collection, a semistructured interview guide (Multimedia Appendix 1) was validated based on 2 interviews with 1 clinician and 1 health technology provider and was additionally critically revised by 1 scientist.

**Table 2:** Composition of participants (N=50).

Main role of participant within the Swiss health care sector	Value, n (%)
Providers of health care services (clinicians, health care organizations, and pharmacies)	9 (18)
Providers of health technologies (medical device industry and health care start-ups)	9 (18)
Health sector associations (innovation-promoting associations and interest groups in the health sector)	7 (14)
Consultancy for health system	7 (14)
Digital health-related experts	5 (10)
Experts in medical informatics and IT^a^	5 (10)
Reimbursement-related actors (insurance and insurance association)	4 (8)
Government- and research-related bodies	4 (8)

^a^IT: information technology.

### Data Analysis

For the data analysis of open-ended questions and comments provided during closed-ended questions, we thematically analyzed the transcripts based on a content analysis method [26] and MAXQDA software (version 11, VERBI GmbH) was used to aid data management. To begin, both investigators closely read each transcript (data orientation). The main investigator then deductively coded one-third of the transcripts and inductively coded for new themes (data reduction). Following the coding, both investigators revised the list of themes, improved codes, and clustered them into categories (data display). Thereafter, the main investigator systematically applied coding to all transcripts. The assistant investigator critically reviewed a sample of 21 coded transcripts (final coding). Finally, both investigators drew on important themes (conclusion drawing).

For the data analysis of closed-ended questions, we applied descriptive statistics. We grouped the 5 scale values of answers into 3 groups (*high to very high*, *medium*, and *low to very low*) and calculated proportions per stakeholder group. Descriptive analyses were conducted using Excel software version 16.16.11 (190619). The data were tabulated.

### Ethical Considerations

This study did not fall within the scope of the Human Research Act. Therefore, authorization from the ethics committee was not required (BASEC-Nr. Req-2019-01070).

## Results

### Characteristics of Interviewees

The sample included stakeholders with different roles in the health sector (Table 3). About half of the participants (26/50, 52%) indicated to have more than 1 professional role in the health sector (see question 1 of Multimedia Appendix 1). Many interviewees (36/50, 72%) believed they had high to very high knowledge of mHealth. Few interviewees (4/50, 8%) thought they had moderate knowledge of mHealth but high knowledge of medical devices in general.

**Table 3:** Characteristics of interviewees.

Main role of participant within the Swiss healthcare sector	Very high to high knowledge of mobile health, n (%)	Average knowledge of mobile health, n (%)	Moderate knowledge of mobile health, n (%)
Providers of health care services (clinicians, health care organizations, and pharmacies)	7 (78)	1 (11)	1 (11)
Providers of health technologies (medical device industry and health care start-ups)	8 (89)	1 (11)	0 (0)
Associations or similar organizations (health and digitization)	5 (72)	1 (14)	1 (14)
Consultancy for health system	5 (71)	2 (29)	0 (0)
Experts in digitization (health and nonhealth sectors)	3 (60)	2 (40)	0 (0)
Experts in medical informatics and IT^a^	3 (60)	2 (40)	0 (0)
Reimbursement-related actors (insurance and insurance association)	2 (50)	1 (25)	1 (25)
Government- and research-related bodies	3 (75)	1 (25)	0 (0)
Total	36 (72)	10 (20)	4 (8)

^a^IT: information technology.

### Potential Relevance and Influence of Mobile Health for the Health System and Health Care Provision

Among the different stakeholder groups, many interviewees believed that mHealth would gain a moderate to high importance in general and for selected aspects of health care provision (Multimedia Appendix 2). Respondents of the group *providers of health services* were relatively reserved about the relevance of mHealth in the Swiss health system compared with other survey groups. Overall, interviewees thought that mHealth would be highly influential for *patient monitoring*. They argued that clinical experience with some mobile solutions is already rising and demonstrated added value to the treatment pathway. Further, they believed that mHealth would be very influential for *diagnostics* because it could be used as a supporting tool for medical decision-making. Many interviewees saw only limited potential for mHealth in the field of *prognosis* of diseases. They believed that the maturity level of the current generation of mHealth technologies was still very low.

Overall, respondents deduced that the integration of mHealth into medical processes will add value to the patient journey. Interviewees highlighted several opportunities and emphasized a wide range of areas to illustrate the potential influence of mHealth on health care provision (Multimedia Appendix 3). Content analysis resulted in 23 topics that we grouped into *patient and patient pathway*, *treatment of disease*, and *diseases and health conditions*.

The number of topics different stakeholder groups focused on varied; *providers of health care services* mentioned a relatively wide range of topics whereas *government- and research-related bodies* emphasized fewer areas. Two or less stakeholder groups brought up the topics: *offering a wider spectrum of care and improving access to health services* and *improving screening options before stationary interventions*.

Many respondents thought that mHealth could have an impact in terms of *improving health literacy and empowerment of patients*, *increasing health care efficiency*, *establishing new preventive care approaches*, *enabling continuous monitoring*, *complementing and supporting traditional treatment concepts*, and *aiding decision-making based on supportive analysis and diagnostics*. Topics that were of less interest to individual interviewees but still mentioned by respondents were *enabling early detection of health risks*, *contributing to outpatient care*, *making therapies simpler, better controllable and less error-prone*, *fostering disease management*, *generating and access to real-life data*, *improving understanding of disease progress*, and *controlling effectiveness of therapies more closely and enabling early detection of adverse or suboptimal response to treatments*.

### Factors Influencing Mobile Health Adoption

Multimedia Appendix 4 shows the results of closed-ended questions regarding the potential relevance of specific determinants for future mHealth adoption. Of the 50 respondents, 1 interviewee had no specific opinion on the determinants of questions 2 and 6 of the interview guide and answered them with *no opinion*. Multimedia Appendix 5 provides a selection of comments obtained during this part of the interviews.

Multimedia Appendix 6 illustrates the topics that respondents highlighted regarding future mHealth adoption based on open-ended questions. Content analysis resulted in 26 topics that we grouped into *trends*, *enablers*, and *restraints*. Multimedia Appendix 7 provides a selection of comments obtained during interviews.

### Trends

Interviewees emphasized a total of 11 topics that we grouped into the categories *changing needs and expectations of patients in the health system*, *rising need for efficient health care delivery*, *growing interest in supporting and optimizing outpatient care*, and *emerging technologies and progressing digitization in the health sector*.

#### Changing Needs and Expectations of Patients in the Health System

Many interviewees concluded that patients are in a phase of upheaval driven by their changing needs and referred to similar changes in the lifestyle area. They considered that mHealth provides patients with a promising opportunity to actively shape this phase. They believed that mHealth adoption would be influenced by patients assuming a more consumer-like mindset, being better informed, and demanding more comprehensive information regarding their health status. In addition, interviewees named the introduction of the electronic patient record and clinicians’ attitudes toward mHealth as important steps toward mHealth adoption. They were convinced that by increasing utilization of digital tools in general and in the health system, people would gradually become accustomed to sharing their health data for medical purposes.

#### Rising Demand for Efficient Health Care Delivery

Many interviewees noted that the rising burden on the health system would drive mHealth acceptance. They believed that health care providers would use mHealth as a strategy to increase process efficiency by integrating it into the patient pathway and responding to changing requirements of the health care environment.

They mentioned that cost savings could be realized through remote follow-up of the patients in both the ambulatory sector by the improved exchange of data with health care providers and the stationary sector by shorter inpatient stays. Yet, no respondent had an opinion about how significant the cost savings may be. Some pointed out that instead of cost savings, new costs may arise. They presumed clinicians would integrate mHealth into their treatment regime in the short term but without adapting traditional workflows. Further, they supposed that patients using mHealth would increase their demand and consumption of health care services in the long term. The driving factors would be new health service opportunities and a growing demand for personalized approaches.

#### Growing Interest in Supporting and Optimizing Outpatient Care

Some interviewees highlighted the *upcoming new opportunities for patients to engage with the health care system*. In addition, they noted that insurance companies and pharmacies showed first attempts to reinvent or adapt their role and business model in their function as health care providers and in response to the digitization in the health system. Respondents emphasized how these 2 players would focus on specific issues that meet the changing needs of patients by utilizing mHealth solutions as a facilitator (eg, preventive care and follow-up advice). Few interviewees highlighted that the role of insurances was controversially discussed among health system stakeholders in general. On the one hand, they had the opportunity to foster health promotion and prevention (introducing awarding programs). On the other hand, they were subject to legal and societal issues (eg, competences and social scoring debate).

Few respondents accentuated that the rising need for integrated solutions in long-term and elderly care would increase the use of mHealth solutions. They felt that the utilization of partially automated and digitally supported health services would be a reasonable solution to meet the growing demand for service and the lack of coordination among caregivers, as well as to finance the resulting costs.

### Enablers

Interviewees emphasized a total of 6 topics that we grouped into categories *growing demand for new financing schemes and incentive concepts for mHealth*, *rising demand for comprehensive information on and stronger body of evidence for mHealth use cases*, and *increasing need for easy to use alternate care approaches*.

#### Growing Demand for New Financing Schemes and Incentive Concepts for Mobile Health

The majority of interviewees maintained that mHealth adoption would significantly depend on new financing schemes and incentive concepts for mHealth that are currently not established in the health system. They named a wide range of ideas about how novel financing concepts would enable and foster mHealth adoption at the ambulatory level. For instance, some highlighted the importance of the capitation model or a new form of diagnostic-related group. They claimed that new schemes and concepts could motivate clinicians to adopt digital health solutions with the aim to increase the efficiency of their treatments. Others mentioned that value- or incentive-based systems for professionals, as seen by the latest developments in the insurance sector for outpatient units, would be a step in the right direction.

Few interviewees envisioned a concept beyond monetary incentives that would increase in importance in the long run due to changing needs and expectations of patients and clinicians. Interviewees presumed that patients might pay mHealth-related costs out of pocket in the long-term once they were convinced that it promotes their health or improves serious health conditions. Further, they believed that doctors would place more emphasis on the quality of the patient-doctor relation for specific medical situations than on monetary benefits.

#### Rising Demand for Comprehensive Information on and Stronger Body of Evidence for Mobile Health Use Cases

Many stakeholders believed that beyond the initial mHealth hype, its adoption is increasingly challenged by a weak body of evidence and thus lack of trust in the promised benefits. Interviewees speculated that potential users would only build up confidence in mHealth when clinicians, in general, are more empowered in the use of digital health tools and understand how it influences their workflow. Some interviewees noted that as long as there was no systematic introduction of mHealth based on clinical studies exploring the value proposition along the treatment pathway, it would not play a significant role in medical services. However, some interviewees also believed that the generation of evidence was challenged by 2 aspects: the rapid development of technology and the lack of recognized methods to assess the clinical value of mHealth use. For instance, few mentioned the need for different evidence standard frameworks for digital health technologies and referred to the United Kingdom. They highlighted the role and influence of research groups and mHealth developers and how they could contribute to a better body of evidence at a disease level.

Further, some interviewees identified the limited information regarding data accuracy and quality (collection and analysis methods used) and noted that it was an important factor for mHealth adoption. They suggested that a quality label or formal body concerned with the quality of mHealth would be necessary to guarantee the quality of mHealth beyond CE certification.

#### Increasing Need for Easy to Use Alternate Care Approaches

Few interviewees focused on the political discussion regarding public funds for inpatient and outpatient care. They purported that if decision-makers would transfer more funds to the area of home-based care, novel care approaches would be fostered. In return, public budgets could be relieved because better and digitally supported care could contribute to stabilize multimorbid patients and prevent avoidable emergencies.

### Restraints

Interviewees emphasized 9 topics in total that we grouped into the categories *rigidness of thinking and siloed actions of health system actors*, *complexity of changing the existing regulations and structures*, *little understanding of mHealth use and the role of clinicians*, and *risk of polarization of population regarding mHealth use*.

#### Rigidness of Thinking and Siloed Actions of Health System Actors

Overall, interviewees mentioned that for successful implementation of mHealth a new way of thinking was needed, but they observed opposite behavior. First, instead of using mHealth as an enabler to contribute to integrative health care approaches, interviewees said that health system actors provided health services in silos. Second, instead of promoting the introduction of digital solutions, interviewees thought that health system actors took opposite measures (eg, difficulties of reimbursement of telemedicine services or the recent cancellation of the poly-medication check provided by pharmacists). Third, instead of developing strategies for new financing schemes, interviewees reported that there was a tendency to force the reimbursement of mHealth into the existing structure. Fourth, instead of fostering the trend to open science (eg, open access to research findings and sharing data) and benefitting from shared data to improve treatments and clinical outcomes, interviewees believed that people were stuck in the data privacy discussion and clinicians were trapped in their habitual management of data.

#### Complexity of Changing the Existing Regulations and Structures

Many interviewees mentioned that unresolved legal issues (eg, liability issues for service providers) and complex regulations may restrain the use of mHealth, and they also referred to the topics listed in Table 1. They indicated that digitization would require an agile mindset, courage, and mutual support. Examples of countries that are digitally more advanced were given to illustrate this while noting the different political frameworks as a major enabler of digitization (eg, Estonia and Singapore). However, interviewees thought that existing regulations should be adapted instead of forcing innovation into structures that have been established for conventional analog approaches.

Furthermore, some interviewees believed that there are still challenging hurdles regarding interconnectivity and IT infrastructure of outpatient and inpatient units limiting the use of mHealth that cannot be solved easily. Moreover, interviewees noted the present inability of health system actors to handle big data, which would be a key leverage point for advanced data analytics.

#### Improving Understanding of Mobile Health Use and the Role of Clinicians

Many interviewees focused on the demarcation of the contribution of clinicians and mHealth to health care provision. Overall, they thought that mHealth could contribute to most settings except for acute care.

Many interviewees believed that despite the wide range of opportunities mHealth applications may provide; many would continue to depend on the clinician’s support and how the patients engage with mHealth solutions (openness and skills). They noted that patients, especially in serious conditions would request face-to-face contact with their treating clinician and would reject services that may create any distance between them and their doctor. In addition, some interviewees supposed that many mHealth solutions for serious diseases would depend on professional instructions and monitoring. Consequently, patients would depend on their clinician’s recommendation and support to use such a tool.

Some interviewees focused on the technological limitations of the present mHealth generation. They were convinced that the level of maturity of mHealth solutions with artificial intelligence-enabled data analysis function was still low. Further, few interviewees feared that counteracting effects regarding people’s health could emerge from increased use of mHealth for monitoring purposes. They expected that people would tend to feel less responsible themselves and provoke health issues.

#### Risk of Polarization of Population Regarding Mobile Health Use and Counter Effects

Many interviewees believed that attitudes of older generations of people toward digital health and diverging digital affinity of people regarding the use of digital solutions in general, would manifest in an uneven mHealth adoption. They believed that a growing fragmentation of health service recipients would be observed in the long-term. For instance, the way people respond to incentive systems that reward healthy behavior and good health conditions. Therefore, healthy people would stay healthier, and people in poorer health would be disadvantaged.

## Discussion

### Principal Findings

The aim of this study is to deepen the understanding of potential mHealth adoption in the Swiss health system. For this, we assessed and evaluated stakeholder perspectives regarding the potential relevance and influence of mHealth for the health system and health care provision, and factors influencing its adoption. The findings will be used to provide an outlook on feasible recommendations for action.

We sought to supplement existing knowledge on enablers and barriers for mHealth use by providing a more differentiated understanding of mHealth adoption in a Swiss context and from different thematic angles that were based on a multifaceted stakeholder perspective. Overall, we found that fostering mHealth adoption is feasible and that it will likely positively influence the health system performance regarding process efficiency and clinical outcome.

We found that mHealth is perceived as a positive development by the large majority of respondents because it could offer multiple opportunities for health care. The respondents believed that mHealth adoption would gradually take place over a longer period and strongly depend on how the patient and physician handle it. Current findings in literature suggest that some areas will likely see more mHealth usage, for example, when it targets telemedicine and patient monitoring [29]. Other areas may evolve slower due to specific requirements and needs of the clinician and patient settings [22], indications, and type of health care utilization.

Our study findings suggest that people have a relatively diverse definition of the added value of mHealth solutions to health care provision. The center of attention of the study participants, which did not include patients, were rather topics that concern health care providers and cost efficiency than topics that could add value to the patient-doctor interface or science (eg, data science). This finding necessitates closer inspection of the patient perspective.

Our study showed that respondents had a high consistency in answering topics that refer to the policy discussion in Switzerland, for instance, regarding access to patient data. However, when interviewees were asked what else will trigger mHealth adoption in Switzerland, new topics were revealed. Respondents paid high attention to changing conditions emerging from societal, technological, environmental, economic, and political domains. This finding is aligned with the current health policy and expert discussions and also with initiatives such as the *Swiss personalized health network* and *Midata* [30]; High attention is addressed to ensure that the patient has access and power over his health data but at the same time fostering a health data sharing culture where data are not owned by profit organizations or enterprises.

The discussed topics are a matter of multiple actors in the health system. The findings suggest that the understanding of future mHealth adoption can be fostered by taking the following aspects into consideration:

- mHealth contribution to bridge the gap between conventional approaches in health care provision and changing conditions will be pivotal for its successful adoption. The systematic introduction of mHealth, a better body of evidence, and the role of novel incentive and financing schemes will be influential.
- The decisive lever will be how well the mHealth solution can build on or connect to existing habits and systems used in medical practice. This has been demonstrated by the examples of mHealth solutions that are already certified by a notified body and used for medical applications and by the findings regarding the Swiss health system.
- Innovative approaches that would imply major digitization steps for health care providers and patients are important but will be less successful in Switzerland in the near future. The high complexity of the Swiss health care system makes it difficult to change existing regulations and structures and at the moment it does not offer the required flexibility to create the necessary framework conditions for such innovations.
- In the triangle of patients, providers, and payers, mHealth adoption is influenced by the implications of the deeply entrenched roles of these actors in health care and their tendency to execute health care provision in a traditional way. In consequence, an innovative mindset and novel health care approaches and settings cannot develop easily.

### Outlook

The digitization hype has led stakeholders to evaluate legal, regulatory, and technological framework conditions across all health system levels to deepen the understanding on how to exploit the potential of emerging technologies and to promote digitization in health [31]. However, as technology is evolving in 3 dimensions (advanced materials, biotechnologies, and digital technologies), health care is experiencing ever greater difficulties in responding to the rapid development of digital solutions. One reason for this is the complexity of changing existing structures in the Swiss health system. On the other hand, health system actors require time to understand the broad spectrum of opportunities for emerging technologies. They have to develop knowledge by reaching out for professional support, which is often lacking.

Findings from other research and recent developments in the health system indicate that discussions and proposals by stakeholders are seeing gradual developments on how to promote mHealth adoption from different angles. For instance, considering social and organizational factors [24] and adopting a holistic approach for the development of digital health solutions [32]. However, a wide range of issues that remain as challenges to mHealth adoption are evolving to increasingly crucial barriers. For instance, solving policy discussions regarding self-determination of patient information, managing digital communication embedded in complex scenarios and treatment pathways, and addressing implications of the rising number of software-based medical devices [33-36].

Digitization in the Swiss health system will take place stepwise as it is an ongoing process of understanding and integrating emerging technology. It highly depends on how the culture of health care actors and patients evolves regarding the adoption and management of digital health solutions [37]. The 2019 commonwealth fund study highlights, for example, that [38]:

- The proportion of Swiss clinicians (69.7%) who document the medical history electronically is still very low.
- Only 46.6% of the Swiss clinicians consider supporting the use of the electronic patient record system.
- A total of 46.5% of Swiss clinicians exchange clinical data of their patients with clinicians from outside of their office.

As long as the level of digitization in the health sector is low, mHealth adoption will progress slowly. We do not only have to manage the expectations regarding added value but also potential drawbacks of the use of novel digital health solutions when they fail as seen recently with a diabetes monitor [39].

On the basis of the findings of our study, recent research, and policy discussion, we reveal an outlook on how mHealth adoption could be better promoted by approaching the topic from new angles and thus beyond the already identified restraints and defined actions:

- Comprehensive information and strong evidence regarding specific mHealth solutions uncovering relevant potentials and limitations.
- Better communication by interest groups and media about the broad application fields of mHealth solutions to support patients, providers, and payers.
- Active patient lobbying to better represent the needs and expectations of patients.
- Strong governance to establish long-term perspectives for the use of digital health technologies and strategies that give the actors room for actions.
- Open discussion and education to overcome barriers that are rooted in the culture of traditional health care along the triangle of patients, providers, and payers.
- Innovative approaches across stakeholders to break down rigid structures and to empower and enable the use and integration of digital health solutions.
- New approaches of cooperation at the interfaces of the triangle of health care recipients, providers, and insurer that provides added value to all involved stakeholders.

### Limitations

Whereas this study contributes to deepening the understanding of factors influencing mHealth adoption in Switzerland, some limitations have to be acknowledged. We used a qualitative method which does not necessarily guarantee the sample being representative for the population of stakeholders involved in the Swiss health system. This study included a variety of stakeholders in terms of expertise and role within the health system, but not all possible interest groups could be considered. Even though patients’ needs and demands are important in the progression of mHealth adoption, they have not been included because their recruitment proved to be very challenging and the focus of this study is on stakeholders that provide health care or shape health care provision (at a system level). Moreover, the sampling was based on a maximum variation strategy and may constitute a selection bias. The interpretation of the findings that not only served to assess enablers and restraints of mHealth adoption but also to define an outlook on how to promote mHealth adoption was a subjective process.

### Conclusions

This study provides an analysis of mHealth adoption in Switzerland from new perspectives. What is becoming increasingly apparent beyond the digital hype, however, is that governance in general and structured data, in particular, are becoming more important. Well-executed health data coordination and exchange are crucial to internalize the added value of new and digitally supported health care environments and ecosystems. The introduction of the Swiss electronic patient record will be an important step forward, but it only provides a formal framework for an advanced playground of health care stakeholders. The adoption of different types of digital health solution is not necessarily disrupting the health system but transforming it to a certain degree. Governance at the different levels of the health system plays a central role in reconciling the different interests of stakeholders and multifaceted impacts that emerge from changing conditions. Digital solutions promise to increase efficiency, contribute to treatment effectiveness, and to improve the mode of communication between patients and health care providers. However, these solutions will not necessarily solve the burden on the system caused by emerging societal needs and changing disease prevalence. Behavioral change of the society and change of habits of stakeholders will also be necessary to internalize the positive effects of digitization. This study provides an outlook on how mHealth adoption could be better promoted by approaching the topic from new angles. Thus, it may contribute to enriching decision-making and actions of policy makers and other stakeholders who have the aim of fostering the adoption of digital solutions into health care.

## Appendix

Multimedia Appendix 1 Semistructured interview guide.

Multimedia Appendix 2 Potential relevance and influence of mobile health.

Multimedia Appendix 3 Topics illustrating the potential influence of mobile health on health care provision.

Multimedia Appendix 4 Potential relevance of selected determinants regarding the future mHealth adoption.

Multimedia Appendix 5 Comments during the collection of attitudes based on closed-ended questions.

Multimedia Appendix 6 Influencing factors for mobile health adoption.

Multimedia Appendix 7 Selection of comments on trends, enablers and restraints obtained during interviews.

## Abbreviations

eHealth: electronic health
mHealth: mobile health
IT: information technology
