# Supplementary material for: Health System Stakeholders’ Perspective on the Role of Mobile Health and Its Adoption in the Swiss Health System: Qualitative Study
Source: JMIR Mhealth Uhealth. 2020 May 11;8(5):e17315. doi: 10.2196/17315 (PMC7248802; doi:10.2196/17315)
Supplement: Multimedia Appendix 1 [file mhealth_v8i5e17315_app1.docx]

| **Expert interview** |
| --- |

| This interview serves to assess viewpoints of pharmacies, health facilities, healthcare professionals, healthcare start-ups, health technology industry, organisations supporting innovation in health, health or accident insurance, federal or cantonal association or initiative or organisation, federal or cantonal health department, digital health-related academics, health-related society or association, consultancy in digital health, expert in digital health data (management and IT), and other experts in digital health regarding their expectations of how mHealth will influence the Swiss healthcare system and their opinions about current and future challenges regarding the adoption and reimbursement of mHealth (classified as medical devices). Thank you for contributing to this research and answering the following questions that will require **approximately 30 minutes**.  If you wish to contact us before or during the process you may contact the following person:     - Main researcher: Dr. Myriam Lingg, independent researcher (E-mail: myriam.lingg@yahoo.de) - Researcher: Dr. Verena Lütschg, independent researcher (E-mail: verena.luetschg@gmail.com) - Collaborator: Prof. Kaspar Wyss, Swiss Tropical and Public Health Institute   Background of the study:   - Mobile health (mHealth) is an integral part of eHealth and refers to a mobile wireless health technology used to collect data e.g. via sensor technology and to store and make the health data available. In healthcare, the aim is to improve the quality and coverage of care, increase access to health information, services and skills, as well as to promote positive changes in healthy behavior to prevent the onset of acute and chronic diseases. - Advances in technologies such as sensors, communication and information systems and analytics empower mHealth based patient monitoring (e.g. physiological parameters) and diagnostic function (e.g. prediction of a level of health risk based on personal data) systems which are expected to support the prevention and diagnosis of diseases. As patient monitoring and diagnostic services become more digital they can take place at home, hospital and outdoors (during activities). - Overall, it opens up new opportunities for the interaction of the population with health services, conventional clinical and preventive care approaches. It is expected to make healthcare more cost-effective and user-friendly. Further, it enables real-time or nearly real-time reporting of diagnostic results to patients and healthcare professionals to elicit rapid and appropriate clinical responses. |
| --- |

**---- To be filled in by the interviewer ----**

| **I. General** |
| --- |

| Archival n°: |  | Site (place / city): |  |
| --- | --- | --- | --- |
|  |  |  |  |
| Interviewer: |  | Date (dd/mm/yy): |  |
|  |  |  |  |
| Start (hh:mm): |  | End (hh:mm): |  |

| **II. Opening and interviewee data** |
| --- |

| 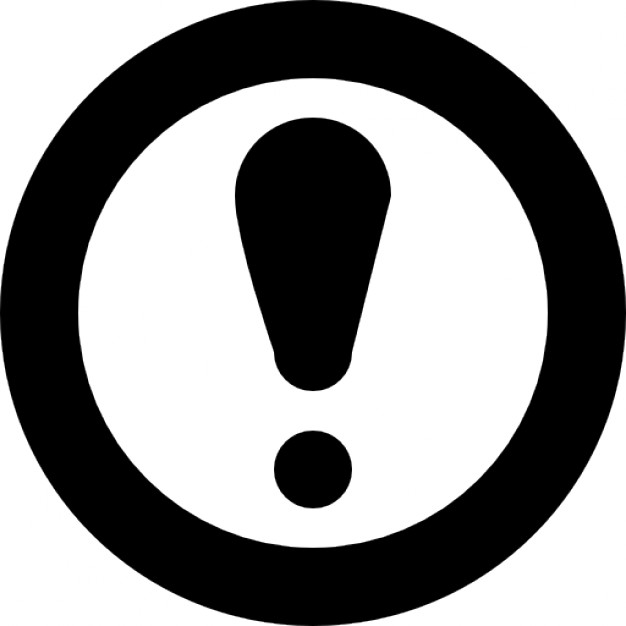 | Please open the interview with a short explanation of the background of the study and the goal of the interview according to the interviewer briefing file. Proceed with the collection of data that describes the interviewee before starting with the semi-structured questionnaire. |
| --- | --- |

**Q II-1**

|  |
| --- |
| Which stakeholder group does the interviewee mainly represent? Please indicate with an X. |

|  | Pharmacy | ☐ |  | Health facility | ☐ |  | Healthcare professional | ☐ |
| --- | --- | --- | --- | --- | --- | --- | --- | --- |
|  |  |  |  |  |  |  |  |  |
|  | Healthcare startup (digital health) | ☐ |  | Health technology industry | ☐ |  | Organisations supporting innovation in health | ☐ |
|  |  |  |  |  |  |  |  |  |
|  | Health, accident and other insurance | ☐ |  | Federal or cantonal association, initiative or organisation | ☐ |  | Cantonal or federal department | ☐ |
|  |  |  |  |  |  |  |  |  |
|  | Academics | ☐ |  | Health-related society or association | ☐ |  | Consultancy in digital health | ☐ |
|  |  |  |  |  |  |  |  |  |
|  | Expert in digital health data | ☐ |  | Other experts for digital health | ☐ |  |  | ☐ |
|  |  |  |  |  |  |  |  |  |
| If other, please specify: |  | | | | | | | |

| **Q II-2** |
| --- |
|  |
| What is the interviewees level of knowledge regarding …? Please indicate with an X one answer per category. |

|  | | |  | Very high |  | High |  | Medium |  | Low |  | Very low |  | No opinion | |
| --- | --- | --- | --- | --- | --- | --- | --- | --- | --- | --- | --- | --- | --- | --- | --- |
|  |  |  | | | | | | | | | | | | |  |
| How familiar are you with the concept of a mHealth application (=store and make health data from a device collecting data and used by the patient)? | | |  | ☐ |  | ☐ |  | ☐ |  | ☐ |  | ☐ |  | ☐ | |
|  | | |  |  |  |  |  |  |  |  |  |  |  |  | |
| How familiar are you with the concept of medical device reimbursement (=how it works in general)? | | |  | ☐ |  | ☐ |  | ☐ |  | ☐ |  | ☐ |  | ☐ | |
|  | | |  |  |  |  |  |  |  |  |  |  |  |  | |
| How familiar are you with the discussion about the reimbursement of mHealth in general? | | |  | ☐ |  | ☐ |  | ☐ |  | ☐ |  | ☐ |  | ☐ | |
|  | | |  |  |  |  |  |  |  |  |  |  |  |  | |

**III. Interview questions**

| **Q III-1** |  |
| --- | --- |
|  |  |
| In your opinion, what role will mHealth play within the Swiss healthcare system in the longterm (5 years and more)? | |

|  |
| --- |

| **Q III-2** |
| --- |
|  |
| How much will the following aspects influence the future use and adoption of mHealth in the Swiss healthcare system? Please indicate with an X your answer per category. |

|  | | |  | Very high |  | High |  | Medium |  | Low |  | Very low |  | No opinion | |
| --- | --- | --- | --- | --- | --- | --- | --- | --- | --- | --- | --- | --- | --- | --- | --- |
|  |  |  | | | | | | | | | | | | |  |
|  | | |  |  |  |  |  |  |  |  |  |  |  |  | |
| Personalisation | | |  | ☐ |  | ☐ |  | ☐ |  | ☐ |  | ☐ |  | ☐ | |
|  | | |  |  |  |  |  |  |  |  |  |  |  |  | |
| Health literacy (patients) | | |  | ☐ |  | ☐ |  | ☐ |  | ☐ |  | ☐ |  | ☐ | |
|  | | |  |  |  |  |  |  |  |  |  |  |  |  | |
| Access to healthcare | | |  | ☐ |  | ☐ |  | ☐ |  | ☐ |  | ☐ |  | ☐ | |
|  | | |  |  |  |  |  |  |  |  |  |  |  |  | |
| Care provider – patient - communication | | |  | ☐ |  | ☐ |  | ☐ |  | ☐ |  | ☐ |  | ☐ | |
|  | | |  |  |  |  |  |  |  |  |  |  |  |  | |
| Access to patient data | | |  | ☐ |  | ☐ |  | ☐ |  | ☐ |  | ☐ |  | ☐ | |
|  | | |  |  |  |  |  |  |  |  |  |  |  |  | |

| **Q III-3** |  |
| --- | --- |
|  |  |
| In your opinion, how will the use of mHealth influence the patient monitoring (e.g. chronic disease management) in the longterm (5 years and more)? | |

|  |
| --- |

| **Q III-4** |  |
| --- | --- |
|  |  |
| In your opinion, how will the use of mHealth influence the patient specific prediction of a disease and its development in the longterm (5 years and more)? | |

|  |
| --- |

| **Q III-5** |  |
| --- | --- |
|  |  |
| In your opinion, how will the use of mHealth influence diagnostic outcomes (e.g. less missed or delayed diagnosis) and in consequence medical decision making? |  |
|  | |

| **Q III-6** |  |
| --- | --- |
|  |  |
| How relevant do you estimate the following aspects for the adoption of mHealth applications in the Swiss healthcare system in the longterm? Please indicate with an X your answer per category. | |

|  | | |  | Very high |  | High |  | Medium |  | Low |  | Very low |  | No opinion | |
| --- | --- | --- | --- | --- | --- | --- | --- | --- | --- | --- | --- | --- | --- | --- | --- |
|  |  |  | | | | | | | | | | | | |  |
|  | | |  |  |  |  |  |  |  |  |  |  |  |  | |
| Obtaining real-time or nearly real-time data of the monitoring of relevant parameters | | |  | ☐ |  | ☐ |  | ☐ |  | ☐ |  | ☐ |  | ☐ | |
|  | | |  |  |  |  |  |  |  |  |  |  |  |  | |
| Ensuring continuity of patient monitoring between two consultations | | |  | ☐ |  | ☐ |  | ☐ |  | ☐ |  | ☐ |  | ☐ | |
|  | | |  |  |  |  |  |  |  |  |  |  |  |  | |
| Enabling advanced diagnostic results through artificial intelligence | | |  | ☐ |  | ☐ |  | ☐ |  | ☐ |  | ☐ |  | ☐ | |
|  | | |  |  |  |  |  |  |  |  |  |  |  |  | |
| Enabling the selection of appropriate therapies due to better diagnostic outputs (reduction of mistakes and failures leading to misdiagnosis) | | |  | ☐ |  | ☐ |  | ☐ |  | ☐ |  | ☐ |  | ☐ | |
|  | | |  |  |  |  |  |  |  |  |  |  |  |  | |
| Shifting the attention of healthcare providers from monitoring to data analysis and interpretation | | |  | ☐ |  | ☐ |  | ☐ |  | ☐ |  | ☐ |  | ☐ | |
|  |  |  | | | | | | | | | | | | |  |
| Providing pilot studies in cooperation with insurance company and healthcare provider | | |  | ☐ |  | ☐ |  | ☐ |  | ☐ |  | ☐ |  | ☐ | |
|  |  |  | | | | | | | | | | | | |  |
| Charging medical fees in the absence of the patient for reviewing patient data collected/provided by mHealth | | |  | ☐ |  | ☐ |  | ☐ |  | ☐ |  | ☐ |  | ☐ | |
|  | | |  |  |  |  |  |  |  |  |  |  |  |  | |

| **Q III-7** |
| --- |
|  |
| How much attention should mHealth developer and provider address to the following aspects? Please indicate with an X your answer per category. |

|  | | |  | Very high |  | High |  | Medium |  | Low |  | Very low |  | No opinion | |
| --- | --- | --- | --- | --- | --- | --- | --- | --- | --- | --- | --- | --- | --- | --- | --- |
|  |  |  | | | | | | | | | | | | |  |
| Proving that the mHealth application is effective regarding patient engagement | | |  | ☐ |  | ☐ |  | ☐ |  | ☐ |  | ☐ |  | ☐ | |
|  |  |  | | | | | | | | | | | | |  |
| Proving that the mHealth application is effective regarding care provider workflow | | |  | ☐ |  | ☐ |  | ☐ |  | ☐ |  | ☐ |  | ☐ | |
|  |  |  | | | | | | | | | | | | |  |
| Proving that the mHealth application is effective regarding the monitoring / diagnostic process | | |  | ☐ |  | ☐ |  | ☐ |  | ☐ |  | ☐ |  | ☐ | |
|  |  |  | | | | | | | | | | | | |  |
| Proving that the mHealth application is useful regarding the monitoring / diagnostic process | | |  | ☐ |  | ☐ |  | ☐ |  | ☐ |  | ☐ |  | ☐ | |
|  |  |  | | | | | | | | | | | | |  |
| Proving that the mHealth application is economical regarding the monitoring / diagnostic process | | |  | ☐ |  | ☐ |  | ☐ |  | ☐ |  | ☐ |  | ☐ | |
|  |  |  | | | | | | | | | | | | |  |

| **Q III-8** |
| --- |
|  |
| How relevant do you estimate the following aspects for the future reimbursement of mHealth in the Swiss healthcare system? Please indicate with an X your answer per category. |

|  | | |  | Very high |  | High |  | Medium |  | Low |  | Very low |  | No opinion | |
| --- | --- | --- | --- | --- | --- | --- | --- | --- | --- | --- | --- | --- | --- | --- | --- |
|  |  |  | | | | | | | | | | | | |  |
| Prescription of mHealth use by healthcare professional as mandatory requirement prior to reimbursement | | |  | ☐ |  | ☐ |  | ☐ |  | ☐ |  | ☐ |  | ☐ | |
|  | | |  |  |  |  |  |  |  |  |  |  |  |  | |
| Regulation of cost-effectiveness requirements for mHealth in order to achieve the required listing | | |  | ☐ |  | ☐ |  | ☐ |  | ☐ |  | ☐ |  | ☐ | |
|  | | |  |  |  |  |  |  |  |  |  |  |  |  | |
| Negotiation between mHealth supplier and insurance companies to reimburse it as evidence based alternative or advanced health services package to an existing monitoring standard | | |  | ☐ |  | ☐ |  | ☐ |  | ☐ |  | ☐ |  | ☐ | |
|  | | |  |  |  |  |  |  |  |  |  |  |  |  | |

| **Q III-9** |  |
| --- | --- |
|  |  |
| Which aspects will be key for the successful adoption of mHealth (for monitoring/diagnostic purposes) in the Swiss healthcare system in the longterm? Please indicate with an X your answer per category. | |

|  | | |  | Very high |  | High |  | Medium |  | Low |  | Very low |  | No opinion | |
| --- | --- | --- | --- | --- | --- | --- | --- | --- | --- | --- | --- | --- | --- | --- | --- |
|  |  |  | | | | | | | | | | | | |  |
| “out of pocket payment” for the use of mHealth | | |  | ☐ |  | ☐ |  | ☐ |  | ☐ |  | ☐ |  | ☐ | |
|  | | |  |  |  |  |  |  |  |  |  |  |  |  | |
| “collective reimbursement” for the use of mHealth | | |  | ☐ |  | ☐ |  | ☐ |  | ☐ |  | ☐ |  | ☐ | |
|  | | |  |  |  |  |  |  |  |  |  |  |  |  | |
| “selective reimbursement” for the use of mHealth | | |  | ☐ |  | ☐ |  | ☐ |  | ☐ |  | ☐ |  | ☐ | |

| **Q III-10** |
| --- |
|  |
| In your opinion, what else will trigger the adoption and reimbursement of mHealth in in the Swiss healthcare system? |

|  |
| --- |
