# Supplementary material for: Health System Stakeholders’ Perspective on the Role of Mobile Health and Its Adoption in the Swiss Health System: Qualitative Study
Source: JMIR Mhealth Uhealth. 2020 May 11;8(5):e17315. doi: 10.2196/17315 (PMC7248802; doi:10.2196/17315)
Supplement: Multimedia Appendix 2 [file mhealth_v8i5e17315_app2.docx]

**Multimedia appendix 2.** Potential relevance and influence of mobile health.

| Categories of relevance and influence of mHealth | Providers of health care services | Suppliers of health technologies | Health sector associations | Consultancy for health system | Experts in digitization | Experts in medical informatics and IT^a^ | Reimbursement-related actors | Government- and research-related bodies |
| --- | --- | --- | --- | --- | --- | --- | --- | --- |
| Potential relevance in general | moderate to high ^b^ | moderate to high | high^c^ | high | moderate to high | high | high | moderate |
| Potential influence regarding *patient monitoring* | moderate to high | high | high | high | high | high | high | high |
| Potential influence regarding *disease prognosis* | moderate | moderate to high | high | moderate to high | high | moderate to high | moderate to high | Moderate to high |
| Potential influence regarding *diagnostics* | moderate to high | high | high | moderate to high | high | moderate to high | moderate to high | high |

^a^IT: information technologies
