# Supplementary material for: Health System Stakeholders’ Perspective on the Role of Mobile Health and Its Adoption in the Swiss Health System: Qualitative Study
Source: JMIR Mhealth Uhealth. 2020 May 11;8(5):e17315. doi: 10.2196/17315 (PMC7248802; doi:10.2196/17315)
Supplement: Multimedia Appendix 3 [file mhealth_v8i5e17315_app3.docx]

**Multimedia appendix 3.** Topics illustrating the potential influence of mobile health on health care provision.

| Identified themes | | Providers of health care services | Suppliers of health technologies | Health sector associations | Consultancy for health system | Experts in digitization | Experts in medical informatics and IT^a^ | Reimbursement-related actors | Government- and research-related bodies |
| --- | --- | --- | --- | --- | --- | --- | --- | --- | --- |
| **Patient and patient pathway** | | | | | | | | | |
|  | Improving health literacy and empowerment of patients | X^b^ | X^b^ | X^b^ | -^c^ | X^b^ | - ^c^ | X^b^ | X^b^ |
|  | Improving patient compliance and responsibility | - ^c^ | X^b^ | X^b^ | - ^c^ | - ^c^ | - ^c^ | - ^c^ | X^b^ |
|  | Generating higher feeling of security | X^b^ | - ^c^ | - ^c^ | - ^c^ | - ^c^ | - ^c^ | X^b^ | - ^c^ |
|  | Increasing quality of life of chronic patients | X^b^ | - ^c^ | X^b^ | - ^c^ | - ^c^ | - ^c^ | X^b^ | - ^c^ |
|  | Stopping disease progress | X^b^ | - ^c^ | - ^c^ | - ^c^ | - ^c^ | - ^c^ | - ^c^ | - ^c^ |
|  | Enabling early detection of health risks | X^b^ | - ^c^ | X^b^ | X^b^ | X^b^ | X^b^ | X^b^ | - ^c^ |
|  | Improving communication and interaction between patient and health care provider | X^b^ | - ^c^ | X^b^ | X^b^ | X^b^ | X^b^ | - ^c^ | X^b^ |
| **Treatment of diseases** | | | | | | | | | |
|  | Increasing health care efficiency | X^b^ | X^b^ | X^b^ | X^b^ | X^b^ | X^b^ | X^b^ | X^b^ |
|  | Offering a wider spectrum of care and improving access to health services | - ^c^ | - ^c^ | - ^c^ | - ^c^ | - ^c^ | - ^c^ | X^b^ | - ^c^ |
|  | Establishing new preventive care approaches | X^b^ | - ^c^ | X^b^ | X^b^ | X^b^ | X^b^ | X^b^ | X^b^ |
|  | Contributing to outpatient care | X^b^ | - ^c^ | X^b^ | - ^c^ | X^b^ | X^b^ | X^b^ | X^b^ |
|  | Improving screening options before stationary interventions | - ^c^ | X^b^ | - ^c^ | X^b^ | - ^c^ | - ^c^ | - ^c^ | - ^c^ |
|  | Making therapies simpler, better controllable, and less error prone | X^b^ | X^b^ | X^b^ | X^b^ | - ^c^ | - ^c^ | - ^c^ | - ^c^ |
|  | Enabling continuous monitoring | X^b^ | X^b^ | X^b^ | X^b^ | X^b^ | X^b^ | X^b^ | X^b^ |
|  | Strengthening continuity of treatments | X^b^ | - ^c^ | - ^c^ | - | - ^c^ | - ^c^ | - ^c^ | - ^c^ |
|  | Reacting faster and more precise on health issues | X^b^ | X^b^ | X^b^ | X^b^ | - ^c^ | - ^c^ | X^b^ | - ^c^ |
|  | Fostering disease management (targeted treatments and follow-up) | X^b^ | X^b^ | - ^c^ | X^b^ | X^b^ | - ^c^ | X^b^ | X^b^ |
|  | Complementing and supporting traditional treatment concepts (eg, by new insights or automation) | X^b^ | X^b^ | X^b^ | X^b^ | X^b^ | X^b^ | X^b^ | X^b^ |
|  | Aiding to decision-making based on supportive analysis and diagnostics | X^b^ | X^b^ | X^b^ | X^b^ | X^b^ | X^b^ | - ^c^ | - ^c^ |
| **Diseases and health conditions** | | | | | | | | | |
|  | Generating and having access to real-life data | X^b^ | X^b^ | X^b^ | X^b^ | - ^c^ | X^b^ | X^b^ | - ^c^ |
|  | Deepening disease insights | X^b^ | X^b^ | - ^c^ | X^b^ | X^b^ | X^b^ | - ^c^ | - ^c^ |
|  | Improving understanding of disease progress | X^b^ | X^b^ | X^b^ | - ^c^ | X^b^ | X^b^ | - ^c^ | - ^c^ |
|  | Controlling the effectiveness of therapies more closely and enabling early detection of adverse or suboptimal response to treatments | X^b^ | X^b^ | X^b^ | X^b^ | X^b^ | X^b^ | X^b^ | - ^c^ |

^a^IT: information technologies

^b^X: respondents put emphasis on this topic to illustrate the potential influence of mHealth on health care provision

^c^-: respondents did not put emphasis on this topic to illustrate the potential influence of mHealth on health care provision.
