# Supplementary material for: Health System Stakeholders’ Perspective on the Role of Mobile Health and Its Adoption in the Swiss Health System: Qualitative Study
Source: JMIR Mhealth Uhealth. 2020 May 11;8(5):e17315. doi: 10.2196/17315 (PMC7248802; doi:10.2196/17315)
Supplement: Multimedia Appendix 4 [file mhealth_v8i5e17315_app4.docx]

Multimedia Appendix 4 - Potential relevance of selected determinants regarding the future mHealth adoption

|  | All stakeholders | Providers of healthcare services | Suppliers of health technologies | Health sector associations | Consultancy for health  system | Experts in digitization | Experts in medical informatics and IT | Reimbursement related actors | Government and research related bodies |
| --- | --- | --- | --- | --- | --- | --- | --- | --- | --- |
|  | N (%) | N (%) | N (%) | N (%) | N (%) | N (%) | N (%) | N (%) | N (%) |
|  |  |  |  |  |  |  |  |  |  |
| **‘Patient’** |  |  |  |  |  |  |  |  |  |
| **Personalization** |  |  |  |  |  |  |  |  |  |
| Very relevant to relevant | 38 (76) | 6 (67) | 8 (89) | 7 (100) | 7 (100) | 3 (60) | 4 (80) | 2 (50) | 1 (25) |
| Medium relevant | 7 (14) | 3 (33) | 1 (11) | 0 (0) | 0 (0) | 0 (0) | 1 (20) | 1 (25) | 1 (25) |
| Little to very little relevant | 3 (6) | 0 (0) | 0 (0) | 0 (0) | 0 (0) | 2 (40) | 0 (0) | 0 (0) | 1 (25) |
| No opinion | 2 (4) | 0 (0) | 0 (0) | 0 (0) | 0 (0) | 0 (0) | 0 (0) | 1 (25) | 1 (25) |
| **Health literacy** |  |  |  |  |  |  |  |  |  |
| Very relevant to relevant | 30 (60) | 5 (56) | 5 (56) | 5 (72) | 5 (71) | 4 (80) | 3 (60) | 2 (50) | 1 (25) |
| Medium relevant | 16 (32) | 4 (44) | 3 (33) | 1 (14) | 2 (29) | 1 (20) | 2 (40) | 2 (50) | 1 (25) |
| Little to very little relevant | 3 (6) | 0 (0) | 1 (11) | 1 (14) | 0 (0) | 0 (0) | 0 (0) | 0 (0) | 1 (25) |
| No opinion | 1 (2) | 0 (0) | 0 (0) | 0 (0) | 0 (0) | 0 (0) | 0 (0) | 0 (0) | 1 (25) |
| **Access to healthcare** |  |  |  |  |  |  |  |  |  |
| Very relevant to relevant | 35 (70) | 5 (56) | 7 (78) | 3 (43) | 6 (86) | 2 (49) | 5 (100) | 4 (100) | 3 (75) |
| Medium relevant | 11 (22) | 4 (44) | 2 (22) | 4 (57) | 1 (14) | 0 (0) | 0 (0) | 0 (0) | 0 (0) |
| Little to very little relevant | 3 (6) | 0 (0) | 0 (0) | 0 (0) | 0 (0) | 3 (60) | 0 (0) | 0 (0) | 0 (0) |
| No opinion | 1 (2) | 0 (0) | 0 (0) | 0 (0) | 0 (0) | 0 (0) | 0 (0) | 0 (0) | 1 (25) |
| **Healthcare provider – patient – communication** |  |  |  |  |  |  |  |  |  |
| Very relevant to relevant | 32 (64) | 4 (44) | 7 (78) | 6 (86) | 4 (57) | 3 (60) | 3 (60) | 2 (50) | 3 (75) |
| Medium relevant | 13 (26) | 3 (34) | 2 (22) | 1 (14) | 2 (28) | 1 (20) | 2 (40) | 2 (50) | 0 (0) |
| Little to very little relevant | 4 (8) | 2 (22) | 0 (0) | 0 (0) | 1 (14) | 1 (20) | 0 (0) | 0 (0) | 0 (0) |
| No opinion | 1 (2) | 0 (0) | 0 (0) | 0 (0) | 0 (0) | 0 (0) | 0 (0) | 0 (0) | 1 (25) |
|  |  |  |  |  |  |  |  |  |  |
| **‘Healthcare provider’** |  |  |  |  |  |  |  |  |  |
| **Access to patient data** |  |  |  |  |  |  |  |  |  |
| Very relevant to relevant | 40 (80) | 7 (78) | 9 (100) | 7 (100) | 7 (100) | 5 (100) | 3 (60) | 0 (0) | 2 (50) |
| Medium relevant | 6 (12) | 2 (22) | 0 (0) | 0 (0) | 0 (0) | 0 (0) | 1 (20) | 3 (75) | 0 (0) |
| Little to very little relevant | 3 (6) | 0 (0) | 0 (0) | 0 (0) | 0 (0) | 0 (0) | 1 (20) | 1 (25) | 1 (25) |
| No opinion | 1 (2) | 0 (0) | 0 (0) | 0 (0) | 0 (0) | 0 (0) | 0 (0) | 0 (0) | 1 (25) |
| **Obtaining real-time or nearly real-time data of the monitoring of relevant parameters** |  |  |  |  |  |  |  |  |  |
| Very relevant to relevant | 40 (80) | 8 (89) | 7 (78) | 6 (86) | 6 (86) | 5 (100) | 3 (60) | 4 (100) | 1 (25) |
| Medium relevant | 7 (14) | 1 (11) | 1 (11) | 1 (14) | 1 (14) | 0 (0) | 2 (40) | 0 (0) | 1 (25) |
| Little to very little relevant | 2 (4) | 0 (0) | 1 (11) | 0 (0) | 0 (0) | 0 (0) | 0 (0) | 0 (0) | 1 (25) |
| No opinion | 1 (2) | 0 (0) | 0 (0) | 0 (0) | 0 (0) | 0 (0) | 0 (0) | 0 (0) | 1 (25) |
| **Ensuring continuity of patient monitoring between two consultations** |  |  |  |  |  |  |  |  |  |
| Very relevant to relevant | 46 (92) | 8 (89) | 8 (89) | 6 (86) | 7 (100) | 5 (100) | 5 (100) | 4 (100) | 3 (75) |
| Medium relevant | 2 (4) | 0 (0) | 1 (11) | 1 (14) | 0 (0) | 0 (0) | 0 (0) | 0 (0) | 0 (0) |
| Little to very little relevant | 1 (2) | 1 (11) | 0 (0) | 0 (0) | 0 (0) | 0 (0) | 0 (0) | 0 (0) | 0 (0) |
| No opinion | 1 (2) | 0 (0) | 0 (0) | 0 (0) | 0 (0) | 0 (0) | 0 (0) | 0 (0) | 1 (25) |
| **Enabling advanced diagnostic results through artificial intelligence** |  |  |  |  |  |  |  |  |  |
| Very relevant to relevant | 38 (76) | 7 (76) | 7 (76) | 7 (100) | 6 (86) | 2 (40) | 4 (80) | 2 (50) | 3 (75) |
| Medium relevant | 8 (16) | 2 (24) | 2 (24) | 0 (0) | 1 (14) | 2 (40) | 0 (0) | 1 (25) | 0 (0) |
| Little to very little relevant | 3 (6) | 0 (0) | 0 (0) | 0 (0) | 0 (0) | 1 (20) | 1 (20) | 1 (25) | 0 (0) |
| No opinion | 1 (2) | 0 (0) | 0 (0) | 0 (0) | 0 (0) | 0 (0) | 0 (0) | 0 (0) | 1 (25) |
| **Enabling the selection of appropriate therapies due to better diagnostic outputs (reduction of mistakes and failures leading to misdiagnosis)** |  |  |  |  |  |  |  |  |  |
| Very relevant to relevant | 39 (78) | 6 (67) | 8 (88) | 7 (100) | 4 (57) | 4 (80) | 4 (80) | 3 (75) | 3 (75) |
| Medium relevant | 8 (16) | 2 (22) | 0 (0) | 0 (0) | 3 (43) | 1 (20) | 1 (20) | 1 (25) | 0 (0) |
| Little to very little relevant | 2 (4) | 1 (11) | 1 (12) | 0 (0) | 0 (0) | 0 (0) | 0 (0) | 0 (0) | 0 (0) |
| No opinion | 1 (2) | 0 (0) | 0 (0) | 0 (0) | 0 (0) | 0 (0) | 0 (0) | 0 (0) | 1 (25) |
| **Shifting the attention of healthcare providers from monitoring to data analysis and interpretation** |  |  |  |  |  |  |  |  |  |
| Very relevant to relevant | 26 (52) | 4 (44) | 5 (56) | 5 (72) | 3 (43) | 2 (40) | 3 (60) | 2 (50) | 2 (50) |
| Medium relevant | 5 (10) | 1 (12) | 0 (0) | 1 (14) | 0 (0) | 1 (20) | 1 (20) | 1 (25) | 0 (0) |
| Little to very little relevant | 16 (32) | 4 (44) | 4 (44) | 1 (14) | 3 (43) | 1 (20) | 1 (20) | 1 (25) | 1 (25) |
| No opinion | 3 (6) | 0 (0) | 0 (0) | 0 (0) | 1 (14) | 1 (20) | 0 (0) | 0 (0) | 1 (25) |
| **Providing pilot studies in cooperation with insurance company and healthcare provider** |  |  |  |  |  |  |  |  |  |
| Very relevant to relevant | 35 (70) | 9 (100) | 6 (67) | 3 (43) | 4 (57) | 5 (100) | 2 (40) | 4 (100) | 2 (50) |
| Medium relevant | 10 (20) | 0 (0) | 2 (22) | 3 (43) | 3 (43) | 0 (0) | 2 (40) | 0 (0) | 0 (0) |
| Little to very little relevant | 3 (6) | 0 (0) | 0 (0) | 1 (14) | 0 (0) | 0 (0) | 1 (20) | 0 (0) | 1 (25) |
| No opinion | 2 (4) | 0 (0) | 1 (11) | 0 (0) | 0 (0) | 0 (0) | 0 (0) | 0 (0) | 1 (25) |
|  |  |  |  |  |  |  |  |  |  |
| **‘mHealth supplier’** |  |  |  |  |  |  |  |  |  |
| **Proving that the mHealth application is effective regarding patient engagement** |  |  |  |  |  |  |  |  |  |
| Very relevant to relevant | 42 (84) | 8 (89) | 8 (89) | 6 (86) | 6 (86) | 5 (100) | 3 (60) | 3 (75) | 3 (75) |
| Medium relevant | 7 (14) | 1 (11) | 1 (11) | 1 (14) | 1 (14) | 0 (0) | 2 (40) | 0 (0) | 1 (25) |
| Little to very little relevant | 1 (2) | 0 (0) | 0 (0) | 0 (0) | 0 (0) | 0 (0) | 0 (0) | 1 (25) | 0 (0) |
| No opinion | 0 (0) | 0 (0) | 0 (0) | 0 (0) | 0 (0) | 0 (0) | 0 (0) | 0 (0) | 0 (0) |
| **Proving that the mHealth application is effective regarding care provider workflow** |  |  |  |  |  |  |  |  |  |
| Very relevant to relevant | 44 (88) | 7 (78) | 8 (89) | 5 (71) | 7 (100) | 5 (100) | 4 (80) | 4 (100) | 4 (100) |
| Medium relevant | 4 (8) | 1 (11) | 0 (0) | 2 (29) | 0 (0) | 0 (0) | 1 (20) | 0 (0) | 0 (0) |
| Little to very little relevant | 2 (4) | 1 (11) | 1 (11) | 0 (0) | 0 (0) | 0 (0) | 0 (0) | 0 (0) | 0 (0) |
| No opinion | 0 (0) | 0 (0) | 0 (0) | 0 (0) | 0 (0) | 0 (0) | 0 (0) | 0 (0) | 0 (0) |
| **Proving that the mHealth application is effective regarding the monitoring / diagnostic process** |  |  |  |  |  |  |  |  |  |
| Very relevant to relevant | 49 (98) | 9 (100) | 9 (100) | 6 (86) | 7 (100) | 5 (100) | 5 (100) | 4 (100) | 4 (100) |
| Medium relevant | 1 (2) | 0 (0) | 0 (0) | 1 (14) | 0 (0) | 0 (0) | 0 (0) | 0 (0) | 0 (0) |
| Little to very little relevant | 0 (0) | 0 (0) | 0 (0) | 0 (0) | 0 (0) | 0 (0) | 0 (0) | 0 (0) | 0 (0) |
| No opinion | 0 (0) | 0 (0) | 0 (0) | 0 (0) | 0 (0) | 0 (0) | 0 (0) | 0 (0) | 0 (0) |
| **Proving that the mHealth application is useful regarding the monitoring / diagnostic process** |  |  |  |  |  |  |  |  |  |
| Very relevant to relevant | 49 (98) | 9 (100) | 9 (100) | 7 (100) | 6 (86) | 5 (100) | 5 (100) | 4 (100) | 4 (100) |
| Medium relevant | 1 (2) | 0 (0) | 0 (0) | 0 (0) | 1 (14) | 0 (0) | 0 (0) | 0 (0) | 0 (0) |
| Little to very little relevant | 0 (0) | 0 (0) | 0 (0) | 0 (0) | 0 (0) | 0 (0) | 0 (0) | 0 (0) | 0 (0) |
| No opinion | 0 (0) | 0 (0) | 0 (0) | 0 (0) | 0 (0) | 0 (0) | 0 (0) | 0 (0) | 0 (0) |
| **Proving that the mHealth application is economical regarding the monitoring / diagnostic process** |  |  |  |  |  |  |  |  |  |
| Very relevant to relevant | 43 (86) | 8 (89) | 7 (78) | 6 (86) | 7 (100) | 5 (100) | 3 (60) | 4 (100) | 3 (75) |
| Medium relevant | 6 (12) | 1 (11) | 2 (22) | 1 (14) | 0 (0) | 0 (0) | 1 (20) | 0 (0) | 1 (25) |
| Little to very little relevant | 1 (2) | 0 (0) | 0 (0) | 0 (0) | 0 (0) | 0 (0) | 1 (20) | 0 (0) | 0 (0) |
| No opinion | 0 (0) | 0 (0) | 0 (0) | 0 (0) | 0 (0) | 0 (0) | 0 (0) | 0 (0) | 0 (0) |
|  |  |  |  |  |  |  |  |  |  |
| **‘Cost reimbursement’** |  |  |  |  |  |  |  |  |  |
| **Charging medical fees in the absence of the patient for reviewing patient data collected/provided by mHealth** |  |  |  |  |  |  |  |  |  |
| Very relevant to relevant | 38 (76) | 6 (67) | 7 (78) | 6 (86) | 7 (100) | 3 (60) | 5 (100) | 2 (50) | 2 (50) |
| Medium relevant | 4 (8) | 3 (33) | 0 (0) | 0 (0) | 0 (0) | 1 (20) | 0 (0) | 0 (0) | 0 (0) |
| Little to very little relevant | 5 (10) | 0 (0) | 0 (0) | 1 (14) | 0 (0) | 1 (20) | 0 (0) | 2 (50) | 1 (25) |
| No opinion | 3 (6) | 0 (0) | 2 (22) | 0 (0) | 0 (0) | 0 (0) | 0 (0) | 0 (0) | 1 (25) |
| **Prescription of mHealth use by healthcare professional as mandatory requirement prior to reimbursement** |  |  |  |  |  |  |  |  |  |
| Very relevant to relevant | 38 (76) | 9 (100) | 5 (56) | 5 (72) | 6 (86) | 5 (100) | 2 (40) | 2 (50) | 4 (100) |
| Medium relevant | 2 (4) | 0 (0) | 0 (0) | 0 (0) | 0 (0) | 0 (0) | 1 (20) | 1 (25) | 0 (0) |
| Little to very little relevant | 8 (16) | 0 (0) | 3 (33) | 2 (28) | 1 (14) | 0 (0) | 1 (20) | 1 (25) | 0 (0) |
| No opinion | 2 (4) | 0 (0) | 1 (11) | 0 (0) | 0 (0) | 0 (0) | 1 (20) | 0 (0) | 0 (0) |
| **Regulation of cost-effectiveness requirements for mHealth in order to achieve the required listing** |  |  |  |  |  |  |  |  |  |
| Very relevant to relevant | 41 (82) | 8 (89) | 6 (67) | 5 (72) | 7 (100) | 5 (100) | 4 (80) | 3 (75) | 3 (75) |
| Medium relevant | 2 (4) | 0 (0) | 0 (0) | 1 (14) | 0 (0) | 0 (0) | 0 (0) | 0 (0) | 1 (25) |
| Little to very little relevant | 4 (8) | 1 (11) | 2 (22) | 0 (0) | 0 (0) | 0 (0) | 0 (0) | 1 (25) | 0 (0) |
| No opinion | 3 (6) | 0 (0) | 1 (11) | 1 (14) | 0 (0) | 0 (0) | 1 (20) | 0 (0) | 0 (0) |
| **Negotiation between mHealth supplier and insurance companies to reimburse it as evidence-based alternative or advanced health services package to an existing monitoring standard** |  |  |  |  |  |  |  |  |  |
| Very relevant to relevant | 36 (72) | 6 (67) | 6 (67) | 6 (86) | 4 (58) | 4 (80) | 3 (60) | 4 (100) | 3 (75) |
| Medium relevant | 10 (20) | 1 (11) | 2 (22) | 1 (14) | 3 (42) | 1 (20) | 1 (20) | 0 (0) | 1 (25) |
| Little to very little relevant | 1 (2) | 1 (11) | 0 (0) | 0 (0) | 0 (0) | 0 (0) | 0 (0) | 0 (0) | 0 (0) |
| No opinion | 3 (6) | 1 (11) | 1 (11) | 0 (0) | 0 (0) | 0 (0) | 1 (20) | 0 (0) | 0 (0) |
| **mHealth expenses covered by out of pocket payments** |  |  |  |  |  |  |  |  |  |
| Very relevant to relevant | 8 (16) | 2 (22) | 1 (11) | 1 (14) | 0 (0) | 1 (20) | 0 (0) | 1 (25) | 2 (50) |
| Medium relevant | 10 (20) | 2 (22) | 0 (0) | 0 (0) | 2 (29) | 1 (20) | 2 (40) | 2 (50) | 1 (25) |
| Little to very little relevant | 29 (58) | 4 (45) | 8 (89) | 6 (86) | 3 (42) | 3 (60) | 3 (60) | 1 (25) | 1 (25) |
| No opinion | 3 (6) | 1 (11) | 0 (0) | 0 (0) | 2 (29) | 0 (0) | 0 (0) | 0 (0) | 0 (0) |
| **mHealth expenses covered by basic insurance** |  |  |  |  |  |  |  |  |  |
| Very relevant to relevant | 43 (86) | 8 (89) | 8 (89) | 7 (100) | 4 (57) | 4 (80) | 4 (80) | 4 (100) | 4 (100) |
| Medium relevant | 2 (4) | 0 (0) | 0 (0) | 0 (0) | 1 (14) | 1 (20) | 0 (0) | 0 (0) | 0 (0) |
| Little to very little relevant | 3 (6) | 1 (11) | 1 (11) | 0 (0) | 0 (0) | 0 (0) | 1 (20) | 0 (0) | 0 (0) |
| No opinion | 2 (4) | 0 (0) | 0 (0) | 0 (0) | 2 (29 | 0 (0) | 0 (0) | 0 (0) | 0 (0) |
| **mHealth expenses covered by selective insurance** |  |  |  |  |  |  |  |  |  |
| Very relevant to relevant | 19 (38) | 3 (33) | 3 (33) | 3 (43) | 3 (43) | 4 (80) | 1 (20) | 2 (50) | 0 (0) |
| Medium relevant | 17 (34) | 2 (23) | 4 (44) | 3 (43) | 1 (14) | 1 (20) | 3 (60) | 2 (50) | 1 (25) |
| Little to very little relevant | 11 (22) | 3 (33) | 2 (23) | 1 (14) | 1 (14) | 0 (0) | 1 (20) | 0 (0) | 3 (75) |
| No opinion | 3 (6) | 1 (11) | 0 (0) | 0 (0) | 2 (29) | 0 (0) | 0 (0) | 0 (0) | 0 (0) |
