# Supplementary material for: Health System Stakeholders’ Perspective on the Role of Mobile Health and Its Adoption in the Swiss Health System: Qualitative Study
Source: JMIR Mhealth Uhealth. 2020 May 11;8(5):e17315. doi: 10.2196/17315 (PMC7248802; doi:10.2196/17315)
Supplement: Multimedia Appendix 5 [file mhealth_v8i5e17315_app5.docx]

**Multimedia appendix 5.** Comments during the collection of attitudes based on closed-ended questions.

| Stakeholder | | Comments |
| --- | --- | --- |
| **Determinants regarding the patient: *personalization*** | | |
|  | Health sector associations (M_201908_02_07) ^a^ | “The use of mHealth opens up opportunities towards personalization.” |
|  | Experts in digitization (M_201910_01_04) ^a^ | “Personalization is more achieved in the field of genomics.” |
|  | Suppliers of health technologies (M_201908_01_21) ^a^ | “mHealth is an essential part for personalization ...” |
|  | Experts in medical informatics and IT^b^ (M_201909_01_27) ^a^ | “If you want personalized medicine and this is the future then you need data and mHealth is an excellent way to get this data.” |
| **Determinants regarding the patient: *health literacy*** | | |
|  | Experts in digitization (M_201909_01_25) ^a^ | “The app itself is not sufficient to increase health literacy of patients.” |
|  | Experts in medical informatics and IT (M_201909_01_27) ^a^ | “The stronger the health literacy of people- and the electronic patient record will contribute to the changing mindset of people - the more you will see how the potential of mHealth emerges.” |
|  | Suppliers of health technologies (M_201910_01_16) ^a^ | “… people want to have more control over information and data and ultimately get data sovereignty.” |
|  | Health sector associations (M_201909_02_05) ^a^ | “With the availability of mHealth health literacy will increase even more.” |
| **Determinants regarding the patient: *access to health care*** | | |
|  | Reimbursement-related actors (M_201909_01_20) ^a^ | “Access to healthcare is very important from a comfort perspective even though we have a high density of providers because people don't know what the right provider is for them. In the future mHealth could make concrete suggestions to them via an ecosystem and use cases.” |
|  | Providers of health care services (M_201907_01_27) ^a^ | “Patients increasingly demand more time and comfort and mHealth could help to bridge the gap.” |
|  | Providers of health care services (M_201907_01_24) ^a^ | “The aspect access to healthcare is important when taking into account that the lack in number of general practitioners will increase.” |
|  | Consultancy for health system (M_201907_02_23) ^a^ | “Hospital density is still high which makes the aspect of access less relevant. But if mHealth is of adequate quality it may have an impact irrespectively of high hospital density.” |
| **Determinants regarding the patient: *health care provider-patient communication*** | | |
|  | Consultancy for health system (M_201907_01_16) ^a^ | “Depends on the generation of doctors because the digital skills differ a lot.” |
|  | Providers of health care services (M_201908_01_16) ^a^ | “Moderate influence because mHealth is not replacing anything but supplementing.” |
|  | Suppliers of health technologies (M_201908_01_27) ^a^ | “One of the fears is that with mHealth use the informal communication will disappear. We expect that machines will make less errors. The question is how this and the loss of the human touch will impact healthcare.” |
|  | Experts in digitization (M_201908_01_23) ^a^ | “It will be relevant where patients and doctors have a long-term relationship or where access to face to face consultations are impacted due to transportation or distance.” |
| **Determinants regarding the health care provider: *access to patient data*** | | |
|  | Providers of health care services (M_201907_01_24) ^a^ | “People are still skeptical about this even though they share a lot of private information with a large audience in the internet. They have a completely different attitude towards their health data.” |
|  | Consultancy for health system (M_201907_02_23) ^a^ | “Given that data is reliable and relevant then it will be important.” |
|  | Government- and research-related bodies (M_201909_01_04) ^a^ | “The more it is used the more data flows. The question is where the data flows to.” |
|  | Consultancy for health system (M_201909_01_09) ^a^ | “Great uncertainty if access to patient data has an influence, especially in the short-term.” |
| **Determinants regarding the health care provider: *Obtaining real-time or nearly real-time data of the monitoring of relevant parameters*** | | |
|  | Health sector association (M_201909_02_05) ^a^ | “A distinction must be made between the monitoring of critically ill patients (important) and prevention (less important). But this aspect still depends very much on regulatory aspects.” |
|  | Experts in digitization (M_201910_01_04) ^a^ | “We get the real time data when we know how to use the collected data. The use and availability of data is key.” |
|  | Government- and research-related bodies (M_201908_01_09) ^a^ | “More exciting than the availability of data is the continuity in data collection.” |
|  | Reimbursement-related actors (M_201909_01_20) ^a^ | “The data will be relevant for those who have to work with them.” |
| **Determinants regarding the health care provider: *Ensuring continuity of patient monitoring between 2 consultations*** | | |
|  | Suppliers of health technologies (M_201908_02_20) ^a^ | “In the field of mental health this could become relevant. For instance, schizophrenic patients have relapses and if these relapses could be predicted then early intervention could be executed.” |
|  | Health sector associations (M_201909_02_04) ^a^ | “Patients and relatives may experience psychological relief through better control.” |
|  | Government- and research-related bodies (M_201909_01_30) ^a^ | “It depends strongly on the form in which the doctor receives information. It should allow to draw enhanced conclusions.” |
|  | Providers of health care services (M_201910_01_24) ^a^ | “Monitoring is a difficult term. Patients probably still have too little confidence in a device.” |
| **Determinants regarding the health care provider: *Enabling advanced diagnostic results through artificial intelligence*** | | |
|  | Experts in digitization (M_201908_01_23) ^a^ | “mHealth will not work independently of the doctor in the near future, only in certain cases.” |
|  | Providers of health care services (M_201907_01_27) ^a^ | “In a short time horizon it is rather not realistic because it will depend on the availability of a large data pool and the data access rights.” |
|  | Reimbursement-related actors (M_201909_01_03) ^a^ | “Little relevance because it is more likely to trigger fears among people.” |
|  | Health sector associations (M_201909_01_23) ^a^ | “In this context it will take much more time until AI enabled mHealth solutions work because they need a large amount of data, learning curves and regulatory solutions.” |
| **Determinants regarding the health care provider: *Enabling the selection of appropriate therapies due to better diagnostic outputs (reduction of mistakes and failures leading to misdiagnosis)*** | | |
|  | Providers of health care services (M_201910_01_24) ^a^ | “One studies medicine and has done advanced education to obtain a professional certification. On the other hand, the device is intended to partially replace one. Great doubts that this will work.” |
|  | Consultancy for health system (M_201910_01_22) ^a^ | “This is where the doctor plays the greatest role with his years of experience. In this sense mHealth is more than a support.” |
|  | Providers of health care services (M_201909_01_06) ^a^ | “Personalized and more precise therapy should be made possible and there mHealth will play an important role.” |
|  | Suppliers of health technologies (M_201908_01_15) ^a^ | “In the case of Switzerland patients are diagnosed and supported by technology. But to make a diagnosis in advance without that patient having seen a doctor first will take very long.” |
| **Determinants regarding the health care provider: *Shifting the attention of health care providers from monitoring to data analysis and interpretation*** | | |
|  | Reimbursement-related actors (M_201908_02_15) ^a^ | “The image of a doctor (professional image) will change massively. But the adjustment takes time as with reimbursement and other aspects. The question will also be in which settings care may be provided. Compliance and regulatory framework would have to be adapted.” |
|  | Experts in digitization (M_201907_02_24) ^a^ | “Doctors are not ready yet to outsource monitoring tasks to machines. The question is also how much the doctors trust the AI technology.” |
|  | Suppliers of health technologies (M_201907_01_25) ^a^ | “Acceptance is not yet there because patients do not agree with the fact that a doctor does not have time for them. By using too many digital technologies this effect is intensified and creates more distance between people and the doctor.” |
|  | Experts in medical informatics and IT (M_201908_01_13) ^a^ | “This would require a change in reimbursement. Currently the costs incurred are not covered.” |
| **Determinants regarding the health care provider: *Providing pilot studies in cooperation with insurance company and health care provider*** | | |
|  | Government- and research-related bodies (M_201909_01_30) ^a^ | “The stakeholders are averse to this because they prefer randomized controlled trials. But if the evidence is there then yes, in the sense of ‘healthcare research’” |
|  | Suppliers of health technologies (M_201908_01_21) ^a^ | “Overall, pilot studies don’t receive a lot of attention, especially not by patients.” |
|  | Reimbursement-related actors (M_201909_01_13) ^a^ | “There is a common interest in reducing costs and cooperation should therefore be encouraged.” |
|  | Experts in medical informatics and IT (M_201909_01_18) ^a^ | “Would be nice but currently it is very marginally implemented.” |
| **Determinants regarding cost reimbursement: *Charging medical fees in the absence of the patient for reviewing patient data collected/provided by mobile health*** | | |
|  | Suppliers of health technologies (M_201908_02_21) ^a^ | “What cannot be billed is not interesting.” |
|  | Health sector associations (M_201908_01_20) ^a^ | “Maybe it will be raised differently. For instance, if the patient is no longer a patient but a customer then there will be more service orientation and thus the patient be enabled to get his service where he sees the greatest added value.” |
|  | Consultancy for health system (M_201909_01_09) ^a^ | “Some kind of mechanism is needed to solve the billing question. A payment system based on the number of patients per month and thus per capita payment could be a solution.” |
|  | Providers of health care services (M_201909_01_06) ^a^ | “In the short-term health apps are more likely to result in a cost increase when we don't evaluate it in the context of clinical evidence.” |
| **Determinants regarding cost reimbursement: *Prescription of mobile health use by health care professional as mandatory requirement before reimbursement*** | | |
|  | Government- and research-related bodies (M_201908_01_09) ^a^ | “In the field of chronic diseases, it could work because then the patient trusts the doctor.” |
|  | Health sector associations (M_201908_01_20) ^a^ | “The question is who is allowed to prescribe based on the amount of data available and how this should be managed when the diagnosis is based on machine results.” |
|  | Suppliers of health technologies (M_201908_02_21) ^a^ | “Not necessarily useful when prescriptible and a recommendation by a doctor would be better. Overall, when the medical professional recommends the use of a mHealth then the patient's compliance will increase.” |
|  | Reimbursement-related actors (M_201909_01_03) ^a^ | “Belongs rather to the quality context and in this sense should form part of the quality contract between associations and organizations and providers.” |
| **Determinants regarding cost reimbursement: *Regulation of cost-effectiveness requirements for mobile health in order to achieve the required listing*** | | |
|  | Health sector associations (M_201908_01_20) ^a^ | “Depending on the specific case. This [regulation of cost-effectiveness requirements] must be done in the long-term and this is still missing.” |
|  | Health sector associations (M_201909_02_05) ^a^ | “Relevant if mHealth is really accepted. But mHealth will primarily establish itself in the private sector and supported by companies that take part in health programs.” |
|  | Experts in medical informatics and IT (M_201909_01_18) ^a^ | “In reality, regulation will be relevant and that will not change in the foreseeable future because money rules.” |
|  | Health care and care organization (M_201909_03_04) ^a^ | “The question is what will be really feasible. Spitex has recently elaborated some recommendations [regarding regulation]. The question arises how to balance between rapidness in development and safety of digital devices. How can providers be sure that a mHealth application really does what it is intended for?” |
| **Determinants regarding cost reimbursement: *Negotiation between mobile health supplier and insurance companies to reimburse it as evidence-based alternative or advanced health services package to an existing monitoring standard*** | | |
|  | Suppliers of health technologies (M_201907_01_25) ^a^ | “Indication-specific negotiations are certainly important.” |
|  | Health sector associations (M_201908_01_20) ^a^ | “It probably won't happen because too many stakeholders live well from the status quo and they will do everything to maintain their situation.” |
|  | Experts in digitization (M_201908_01_23) ^a^ | “The market can only be moved by pressure (laws or money) and everything else won't have success.” |
|  | Reimbursement-related actors (M_201909_01_20) ^a^ | “Insurance companies will probably develop their own mHealth solutions targeted to their needs and offering them in an ecosystem that they are going to create.” |

^a^Text in parenthesis: Participant number

^b^IT: information technologies.
