# Supplementary material for: Health System Stakeholders’ Perspective on the Role of Mobile Health and Its Adoption in the Swiss Health System: Qualitative Study
Source: JMIR Mhealth Uhealth. 2020 May 11;8(5):e17315. doi: 10.2196/17315 (PMC7248802; doi:10.2196/17315)
Supplement: Multimedia Appendix 6 [file mhealth_v8i5e17315_app6.docx]

**Multimedia Appendix 6.** Influencing factors for mobile health adoption.

| Influencing factors for mobile health | | | Providers of health care services | Suppliers of health technologies | Health sector associations | Consultancy for health system | Experts in digitization | Experts in medical informatics and information technology (IT) | Reimbursement-related actors | Government- and research-related bodies |
| --- | --- | --- | --- | --- | --- | --- | --- | --- | --- | --- |
| **Trends** | | | | | | | | | | |
|  | **Changing needs and expectations of patients in the health system** | | | | | | | | | |
|  |  | Rising importance of digital tools in all areas of life and slowly growing openness toward digital managed health data | ✓^a^  1**^d^** | ✓^a^  2**^e^** | -^b^ | ✓^a^  0**^c^** | ✓^a^  1**^d^** | ✓^a^  4**^g^** | ✓^a^  2**^e^** | ✓^a^  2**^e^** |
|  |  | Increasing consumer mindset of patients (eg, less waiting time, more participation) | ✓^a^  1**^d^** | ✓^a^  1**^d^** | ✓^a^  1**^d^** | ✓^a^  1**^d^** | ✓^a^  3**^f^** | ✓^a^  1**^d^** | ✓^a^  1**^d^** | ✓^a^  1**^d^** |
|  |  | Better informed patients, rising sense of self-responsibility | ✓^a^  2**^e^** | ✓^a^  3**^f^** | ✓^a^  2**^e^** | ✓^a^  2**^e^** | ✓^a^  3**^f^** | ✓^a^  3**^f^** | ✓^a^  2**^e^** | ✓^a^  3**^f^** |
|  | **Increasing need for efficient health care delivery** | | | | | | | | | |
|  |  | Increasing burden on health system and demand for more efficiency (eg, aging population, rising health care costs, growing shortage of health care professionals) | ✓^a^  2**^e^** | ✓^a^  3**^f^** | ✓^a^  2**^e^** | ✓^a^  1**^d^** | ✓^a^  2**^e^** | ✓^a^  4**^g^** | ✓^a^  3**^f^** | ✓^a^  2**^e^** |
|  |  | Rising demand for better health (eg, preventive care and personalized approaches) | ✓^a^  1**^d^** | ✓^a^  1**^d^** | ✓^a^  3**^f^** | ✓^a^  2**^e^** | ✓^a^  4**^g^** | ✓^a^  3**^f^** | ✓^a^  1**^d^** | ✓^a^  3**^f^** |
|  |  | Increasing demand for efficient health system interactions (eg, avoiding unnecessary medical appointments and delayed treatments, coordination and automatization of treatment steps) | ✓^a^  0**^c^** | ✓^a^  1**^d^** | ✓^a^  2**^e^** | ✓^a^  2**^e^** | ✓^a^  2**^e^** | ✓^a^  2**^e^** | ✓^a^  2**^e^** | ✓^a^  1**^d^** |
|  | **Growing interest in supporting and optimizing outpatient care** | | | | | | | | | |
|  |  | Upcoming new opportunities for patients to engage with the health care system (eg, telemedicine offers, changing business models of insurances and pharmacies) | ✓^a^  0**^c^** | ✓^a^  1**^d^** | ✓^a^  2**^e^** | -^b^ | ✓^a^  3**^f^** | ✓^a^  3**^f^** | ✓^a^  1**^d^** | -^b^ |
|  |  | Growing demand for high quality solutions enabling patients to better deal with diseases and rehabilitative care approaches or programs | ✓^a^  1**^d^** | ✓^a^  2**^e^** | -^b^ | ✓^a^  0**^c^** | ✓^a^  3**^f^** | ✓^a^  2**^e^** | ✓^a^  3**^f^** | ✓^a^  1**^d^** |
|  | **Emerging technologies and progressing digitization in the health sector** | | | | | | | | | |
|  |  | Increasing utilization of emerging technologies (eg, novel sensors) | ✓^a^  0**^c^** | ✓^a^  1**^d^** | ✓^a^  0**^c^** | -^b^ | ✓^a^  2**^e^** | ✓^a^  2**^e^** | ✓^a^  1**^d^** | -^b^ |
|  |  | Rising interest in open science and artificial intelligence-enabled data analysis | ✓^a^  1**^d^** | ✓^a^  3**^f^** | ✓^a^  3**^f^** | ✓^a^  1**^d^** | ✓^a^  3**^f^** | ✓^a^  2**^e^** | ✓^a^  2**^e^** | ✓^a^  2**^e^** |
|  |  | Advancing digitization in health system (eg, electronic patient dossier, vaccination dossier, and telemedicine) | ✓^a^  1**^d^** | ✓^a^  2**^e^** | ✓^a^  1**^d^** | ✓^a^  2**^e^** | ✓^a^  1**^d^** | ✓^a^  1**^d^** | -^b^ | ✓^a^  2**^e^** |
| **Enablers** | | | | | | | | | | |
|  | **Growing need for new financing schemes and incentive concepts for mobile health** | | | | | | | | | |
|  |  | Growing need for clear reimbursement of mHealth use | ✓^a^  2**^e^** | ✓^a^  2**^e^** | ✓^a^  2**^e^** | ✓^a^  1**^d^** | ✓^a^  1**^d^** | ✓^a^  2**^e^** | ✓^a^  3**^f^** | ✓^a^  1**^d^** |
|  |  | Rising demand for new financing concepts (eg, capitation model, reward programs for providers) | ✓^a^  2**^e^** | ✓^a^  2**^e^** | ✓^a^  2**^e^** | ✓^a^  1**^d^** | ✓^a^  3**^f^** | ✓^a^  3**^f^** | ✓^a^  3**^f^** | ✓^a^  4**^g^** |
|  | **Rising demand for comprehensive information on and stronger body of evidence for mobile health use cases** | | | | | | | | | |
|  |  | Providing comprehensive information how to integrate mHealth into patient pathway | ✓^a^  3**^f^** | ✓^a^  1**^d^** | -^b^ | ✓^a^  2**^e^** | ✓^a^  1**^d^** | -^b^ | ✓^a^  3**^f^** | ✓^a^  1**^d^** |
|  |  | Generating strong body of evidence regarding clinical benefits and economic impact on the health care provision | ✓^a^  2**^e^** | ✓^a^  2**^e^** | ✓^a^  3**^f^** | ✓^a^  4**^g^** | ✓^a^  2**^e^** | ✓^a^  2**^e^** | ✓^a^  4**^g^** | ✓^a^  2**^e^** |
|  |  | Guaranteeing quality of mobile health (eg, precision of data and reliable output parameters) | ✓^a^  2**^e^** | ✓^a^  2**^e^** | ✓^a^  1**^d^** | ✓^a^  2**^e^** | ✓^a^  2**^e^** | -^b^ | ✓^a^  1**^d^** | ✓^a^  1**^d^** |
|  | **Increasing need for easy to use alternate care approaches** | | | | | | | | | |
|  |  | Developing integrated care solutions and providing alternate care pathways (eg, of the elderly population) | ✓^a^  0**^c^** | ✓^a^  2**^e^** | ✓^a^  0**^c^** | ✓^a^  0**^c^** | ✓^a^  1**^d^** | ✓^a^  1**^d^** | ✓^a^  2**^e^** | ✓^a^  3**^f^** |
| **Restraints** | | | | | | | | | | |
|  | **Rigidness of thinking and actions of health system actors** | | | | | | | | | |
|  |  | Operating isolated instead of fostering integrative approaches | ✓^a^  3**^f^** | ✓^a^  2**^e^** | ✓^a^  1**^d^** | ✓^a^  2**^e^** | ✓^a^  2**^e^** | ✓^a^  4**^g^** | ✓^a^  1**^d^** | ✓^a^  1**^d^** |
|  |  | Sticking to conventional approaches and structure | ✓^a^  0**^c^** | ✓^a^  3**^f^** | ✓^a^  1**^d^** | ✓^a^  1**^d^** | ✓^a^  3**^f^** | ✓^a^  1**^d^** | ✓^a^  3**^f^** | -^b^ |
|  |  | Remaining data privacy and management discussion | ✓^a^  2**^e^** | ✓^a^  1**^d^** | ✓^a^  2**^e^** | ✓^a^  0**^c^** | ✓^a^  1**^d^** | ✓^a^  3**^f^** | ✓^a^  1**^d^** | ✓^a^  2**^e^** |
|  | **Complexity of changing the existing regulations and structures** | | | | | | | | | |
|  |  | Preventing advances in mobile health adoption due to complexity of health system and legal issues | ✓^a^  0**^c^** | ✓^a^  1**^d^** | ✓^a^  2**^e^** | ✓^a^  3**^f^** | -^b^ | ✓^a^  1**^d^** | ✓^a^  3**^f^** | ✓^a^  2**^e^** |
|  |  | Lacking willingness to change (eg, agile mindset, courage, and mutual support of health system stakeholders) | ✓^a^  0**^c^** | ✓^a^  2**^e^** | ✓^a^  2**^e^** | -^b^ | ✓^a^  3**^f^** | ✓^a^  3**^f^** | ✓^a^  2**^e^** | ✓^a^  4**^g^** |
|  |  | Remaining and new issues regarding interconnectivity and IT^g^ infrastructure | ✓^a^  0**^c^** | ✓^a^  1**^d^** | ✓^a^  0**^c^** | ✓^a^  2**^e^** | ✓^a^  1**^d^** | ✓^a^  2**^e^** | ✓^a^  1**^d^** | ✓^a^  1**^d^** |
|  | **Little understanding of mobile health use and the role of clinicians** | | | | | | | | | |
|  |  | Importance of clinicians´ stake in patient pathway | ✓^a^  2**^e^** | ✓^a^  3**^f^** | ✓^a^  0**^c^** | -^b^ | ✓^a^  1**^d^** | ✓^a^  4**^g^** | ✓^a^  4**^g^** | ✓^a^  2**^e^** |
|  |  | Rising awareness of the limitations of mHealth functionalities | ✓^a^  2**^e^** | ✓^a^  2**^e^** | ✓^a^  2**^e^** | ✓^a^  2**^e^** | ✓  3**^f^** | ✓  4**^g^** | ✓  1**^d^** | ✓  1**^d^** |
|  | **Risk of polarization of population regarding mobile health use** | | | | | | | | | |
|  |  | Restraining effects of age-related and preference-based factors and growing fragmentation of health service recipients | ✓^a^  1**^d^** | ✓^a^  2**^e^** | -^b^ | ✓^a^  2**^e^** | ✓^a^  1**^d^** | -^b^ | ✓^a^  4**^g^** | -^b^ |

^a^✓: Topic mentioned by at least 1 interviewee.

^b^-: Topic mentioned by no interviewee.

**^c^**0: Topic mentioned by very few interviewees (1%-20%).

**^d^**1: Topic mentioned by few interviewees (20%-39%).

**^e^**2: Topic mentioned by many interviewees (40%-59%).

**^f^**3: Topic mentioned by a lot of interviewees (60%-79%).

**^g^**4: Topic mentioned by almost all interviewees (80%- 100%).
