# Supplementary material for: Health System Stakeholders’ Perspective on the Role of Mobile Health and Its Adoption in the Swiss Health System: Qualitative Study
Source: JMIR Mhealth Uhealth. 2020 May 11;8(5):e17315. doi: 10.2196/17315 (PMC7248802; doi:10.2196/17315)
Supplement: Multimedia Appendix 7 [file mhealth_v8i5e17315_app7.docx]

Multimedia appendix 7 - Selection of comments on trends, enablers and restraints obtained during interviews

|  |  |
| --- | --- |
| **Trends - Changing needs and expectations of patients in the health system** | |
|  |  |
| Providers of healthcare services (M_201909_03_04) ^a^ | “The role of professionals towards patients is changing. Healthcare provision must become more transparent because patients demand more participation in their treatment pathway. Through digitization they become more involved.” |
| Health sector associations  (M_201909_02_05) ^a^ | “Today the doctor is the one who informs the patient and works with the data and in the future the patient will come with the data. The data flow will turn around.” |
| Experts in digitization  (M_201907_02_24) ^a^ | “Patients are nowadays consumers and what we observe a lot is that they have very specific consumer expectations regarding which services they expect to get payed and offered.” |
| Experts in medical informatics and IT^b^  (M_201908_01_13) ^a^ | “There is an increase in consumer products like apple watch and the society will start to expect that everybody will be able to handle those tools. This will exert pressure on the health sector. Whether it this will be of great relevance is another question.” |
|  |  |
| **Trends - Increasing need for efficient healthcare delivery** | |
|  |  |
| Providers of healthcare services  (M_201907_01_24) ^a^ | “The use of process solutions such as mHealth will help to ensure that employees can work with the patient themselves as long as possible and spend less time on administrative tasks.” |
| Government and research related bodies  (M_201908_01_09) ^a^ | “mHealth will play a large role if you consider the increasing cost burden of chronic disease. We have to think about the potentials of such digital tools.” |
| Suppliers of health technologies  (M_201908_01_27) ^a^ | “The population is ageing but also the risk adverse population grows. And the economic pressure rises. So mHealth could help to lower the cost.” |
| Experts in digitization  (M_201909_01_25) ^a^ | “Sensors are getting better… doctors will be able to make a much more differentiated diagnosis … this will help to save costs because a treatment can be started earlier.” |
|  |  |
| **Trends - Growing interest in supporting and optimizing outpatient care** | |
|  |  |
| Reimbursement related actors  (M_201909_01_20) ^a^ | “Insurances will use mHealth as a new channel of interaction for information exchange with the patient beyond the evidence-based efficacy of a therapy.” |
| Health sector associations  (M_201908_02_07) ^a^ | “We observe that with mHealth a new category can be created for healthcare to coordinate existing medical offers. The coordination and consumption of health service offers can be enhanced by using mHealth tools.” |
| Consultancy for health system  (M_201909_01_09) ^a^ | “Keyword “hospital at home”. By the adoption of versatile mHealth solutions the provision of health services could be changed in the long-term.” |
| Experts in medical informatics and IT  (M_201909_01_27) ^a^ | “The topic connected devices plays a role here. It is an interesting market for pharmacies. Today, pharmacies are limited to selling only the drugs, but they want to change that. One possibility is to partially replace the family doctor. On the other hand, however, they are also predestined to sell devices to customers. This opens up the opportunity for them to take measures themselves.” |
|  |  |
| **Trends - Emerging technologies and progressing digitization in the health sector** | |
|  |  |
| Providers of healthcare services  (M_201909_01_06) ^a^ | “The demand for the digital vaccination dossier is rising even though it’s an out of pocket payment for the patient. Patients request this service because it provides them with more flexibility such as the vaccination in pharmacies.” |
| Health sector associations  (M_201909_01_05) ^a^ | “The activity of startups is key. They push the development of mHealth in terms of digital therapies and use of integrated sensors. It is the first step towards tech in medicine.” |
| Experts in medical informatics and IT  (M_201907_01_23) ^a^ | “The more the digital tools implement AI the more one will have the possibility to make use of it regarding a deeper understanding of different health states and progress. |
| Government and research related bodies  (M_201908_01_09) ^a^ | “Whether it is mHealth or not will not be decisive. It is all about push technology; using sensors to collect data). We will be able to collect a relatively large amount of data and then understand what we can do with it.” |
|  |  |
| **Enablers - Growing need for new financing schemes and incentive concepts for mHealth** | |
|  |  |
| Experts in digitization  (M_201908_01_23) ^a^ | “Our thinking is anchored too strongly in classic compensation systems like Tarmed and an evidence-based compensation model is necessary so that medical outcome becomes more important...” |
| Experts in medical informatics and IT  M_201909_01_18) ^a^ | “It depends a lot on the respective insurances and if they accept the use of mHealth. Otherwise mHealth adoption will be impaired. Overall it will take at least another five years.” |
| Providers of healthcare services  (M_201907_02_19) ^a^ | “There is a new tendency from insurance companies towards doctors who get offered economic incentives when they integrate digital tools that contribute to quality improvement in their clinical approach – thus the expenses for adopting digital tools is being compensated by such incentives.” |
| Suppliers of health technologies  (M_201908_01_15) ^a^ | “We have to move to a single health insurance system or at least get away from the enormous number of insurance companies to form the same basic layer plus offer specialized business models for specific diseases.” |
|  |  |
| **Enablers - Rising demand for comprehensive information on and stronger body of evidence for mHealth use cases** | |
|  |  |
| Suppliers of health technologies  (M_201907_01_25) ^a^ | “It needs evidence in order to be remunerated. But the execution of those studies is in conflict with rapidly changing technologies.” |
| Providers of healthcare services  (M_201907_01_05) ^a^ | “One important driver will be how well users (patients and doctors) are informed about the mHealth device. Thus, advantages or additional benefits must be clearly evident and it must be shown that it goes beyond the current standard and why this is important.” |
| Reimbursement related actors  (M_201909_01_13) ^a^ | “As long as the medical benefit is not proven it will not prevail and it will take some time to prove it.” |
| Government and research related bodies  (M_201909_01_04) ^a^ | “Healthcare professionals lack the time to inform themselves about high quality and trustworthy digital tools. This needs to be solved. For instance, we will need something like a catalogue including quality criteria...” |
|  |  |
| **Enablers - Increasing need for easy to use alternate care approaches** | |
|  |  |
| Health sector associations  (M_201908_02_07) ^a^ | “The inpatient sector still receives more financial support from the government then the outpatient sector such as home care settings. Care at home has a need for alternate approaches to improve for example the monitoring. This should be promoted like traditional means such as wheelchairs.” |
| Suppliers of health technologies  (M_201908_02_21) ^a^ | “Digital tools have a great impact regarding social trends. The goal must be to supply care as long as possible to people at home and avoid to going to doctors all the time. That can be avoided by regular monitoring and telemedical services.” |
| Suppliers of health technologies  (M_201908_02_20) ^a^ | “Integrating digital tools in the field of care of multi-morbid patients makes sense but who pays for it… what is important is the value of the solution and how all the people involved in a workflow get value from it.” |
| Government and research related bodies  (M_201909_01_30) ^a^ | “This is being investigated by means of studies but what we see is that also in the field of care of elderly people not all actors work together. Scaling up the health applications is the biggest challenge… technology adoption could contribute.” |
|  |  |
| **Restraints - Rigidness of thinking and actions of health system actors** | |
|  |  |
| Experts in digitization  (M_201910_01_04) ^a^ | “Working in silos is very common. Everyone will try to use mHealth but they will work in silos ....” |
| Government and research related bodies  (M_201907_01_30) ^a^ | “This depends strongly on the data protection discussion. Other systems do not have these barriers as much as Switzerland. For example, Scandinavian countries are much more advanced.” |
| Experts in medical informatics and IT  (M_201908_02_16) ^a^ | “It is about the information monopoly. Without transparency you can’t compare a doctor’s performance. The doctor’s culture is equal to an alpha-male and this is a generation problem. The current and older generation of physicians is not interested to learn new things.” |
| Suppliers of health technologies  (M_201908_01_15) ^a^ | “Fragmentation is taking place across all levels in the health system … Hospitals continue to make adaptations on the basis of old IT architectures instead of conducting a complete revision (one takes the path that is least invasive)… Switzerland as a high-tech country that is locked in its own swamp.” |
|  |  |
| **Restraints - Complexity to change existing regulations and structures** | |
|  |  |
| Providers of healthcare services  (M_201907_01_24) ^a^ | “The federalism in Switzerland makes digitization complex.” |
| Experts in digitization  (M_201909_01_25) ^a^ | “We often forget that the only actor who doesn’t have a lobby is the patient. For this reason, mHealth has a very difficult position… The Swiss Patient Organization (SPO) is much too small to have any effect and this can only be changed at a policy level.” |
| Health sector associations  (M_201909_01_23) ^a^ | “Liability law hurdles regarding the doctor’s duty of care will not easily be able to cope with digitization.” |
| Supplier of health technologies  (M_201908_01_15) ^a^ |  |
|  |  |
| **Restraints - Little understanding of mHealth use and the role of clinicians** | |
|  |  |
| Experts in medical informatics and IT  (M_201907_01_23) ^a^ | “However, it is important to distinguish that mHealth interventions will and should only support the doctor and not replace him. We can see this in the current discussions about the legal situation. AI will relieve but not replace.” |
| Experts in digitization  (M_201907_01_19) ^a^ | “With regards to diagnostic results and medical decision making the doctor will remain in his position and be the one making decisions.” |
| Experts in medical informatics and IT  (M_201909_01_18) ^a^ | “In some areas we see good progress for remote and long-term monitoring …However, data collection is still too imprecise and devices are often too bulky to carry.” |
| Consultancy for health system  (M_201910_01_22) ^a^ | “Digitization has a great potential in connection with artificial intelligence. But mHealth alone will not have great potential. It depends on the technological progress.” |
|  |  |
| **Restraints - Risk of polarization of population regarding mHealth use** | |
|  |  |
| Health sector associations  (M_201909_02_09) ^a^ | ”Furthermore, the older generations are often not well experienced in the use of technology. The next generation has more potential. |
| Health sector associations  (M_201909_01_05) ^a^ | “A two-class system for healthcare delivery will be formed.” |
| Supplier of health technologies  (M_201908_01_21) ^a^ | “People have to learn or get familiar to receive the same quality at a distance. That needs a lot of explanation.” |
| Providers of healthcare services  (M_201910_01_24) ^a^ | “… we will tend to make a classification towards reward versus no reward. In consequence, one would have to be ill in order to benefit from a mHealth.” |
|  |  |

^a^Text in parenthesis: Participant number

^b^IT: information technologies.
